# Multi-omics reveal microbial determinants impacting the treatment outcome of antidepressants in major depressive disorder

**DOI:** 10.1186/s40168-023-01635-6

**Published:** 2023-08-28

**Authors:** Yaping Wang, Jingjing Zhou, Junbin Ye, Zuoli Sun, Yi He, Yingxin Zhao, Siyu Ren, Guofu Zhang, Min Liu, Peng Zheng, Gang Wang, Jian Yang

**Affiliations:** 1 Beijing Key Laboratory of Mental Disorders, National Clinical Research Center for Mental Disorders & National Center for Mental Disorders, Beijing Anding Hospital, Capital Medical University, Beijing, 100088 China; 2 Advanced Innovation Center for Human Brain Protection, Capital Medical University, Beijing, 100069 China; 3 Beijing WeGenome Paradigm Co., Ltd, Beijing, China; 4 Department of Neurology, The First Affiliated Hospital of Chongqing Medical University, Chongqing, 400016 China; 5 NHC Key Laboratory of Diagnosis and Treatment On Brain Functional Diseases, The First Affiliated Hospital of Chongqing Medical University, Chongqing, 400016 China

**Keywords:** Major depressive disorder, Gut microbiota, Antidepressants, Sporulation gene

## Abstract

**Background:**

There is a growing body of evidence suggesting that disturbance of the gut-brain axis may be one of the potential causes of major depressive disorder (MDD). However, the effects of antidepressants on the gut microbiota, and the role of gut microbiota in influencing antidepressant efficacy are still not fully understood.

**Results:**

To address this knowledge gap, a multi-omics study was undertaken involving 110 MDD patients treated with escitalopram (ESC) for a period of 12 weeks. This study was conducted within a cohort and compared to a reference group of 166 healthy individuals. It was found that ESC ameliorated abnormal blood metabolism by upregulating MDD-depleted amino acids and downregulating MDD-enriched fatty acids. On the other hand, the use of ESC showed a relatively weak inhibitory effect on the gut microbiota, leading to a reduction in microbial richness and functions. Machine learning-based multi-omics integrative analysis revealed that gut microbiota contributed to the changes in plasma metabolites and was associated with several amino acids such as tryptophan and its gut microbiota-derived metabolite, indole-3-propionic acid (I3PA). Notably, a significant correlation was observed between the baseline microbial richness and clinical remission at week 12. Compared to non-remitters, individuals who achieved remission had a higher baseline microbial richness, a lower dysbiosis score, and a more complex and well-organized community structure and bacterial networks within their microbiota. These findings indicate a more resilient microbiota community in remitters. Furthermore, we also demonstrated that it was not the composition of the gut microbiota itself, but rather the presence of sporulation genes at baseline that could predict the likelihood of clinical remission following ESC treatment. The predictive model based on these genes revealed an area under the curve (AUC) performance metric of 0.71.

**Conclusion:**

This study provides valuable insights into the role of the gut microbiota in the mechanism of ESC treatment efficacy for patients with MDD. The findings represent a significant advancement in understanding the intricate relationship among antidepressants, gut microbiota, and the blood metabolome. Additionally, this study offers a microbiota-centered perspective that can potentially improve antidepressant efficacy in clinical practice. By shedding light on the interplay between these factors, this research contributes to our broader understanding of the complex mechanisms underlying the treatment of MDD and opens new avenues for optimizing therapeutic approaches.

Video Abstract

**Supplementary Information:**

The online version contains supplementary material available at 10.1186/s40168-023-01635-6.

## Background

Major depressive disorder (MDD) is a debilitating mental illness that affects approximately 350 million people worldwide [1, 2]. Selective serotonin reuptake inhibitors (SSRIs) are considered as the first-line treatment option for patients with MDD [3]. However, it is important to note that the remission rate associated with SSRIs can be as low as one-third [4, 5]. Although metabotype and pharmacogenomics have been proposed as potential factors that could contribute to the variability in antidepressant response [6, 7], the exact underlying mechanisms and predictive markers for this heterogeneity have not been fully understood thus far. Therefore, it is imperative to thoroughly investigate the factors associated with the effectiveness of antidepressant treatment in order to enhance the prognosis of patients diagnosed with MDD.

Multiple studies conducted by our research team, as well as other researchers, have indicated that the dysfunction of the gut-brain axis could potentially contribute to the development of MDD. This dysfunction is associated with various metabolic changes both in the periphery and central nervous systems. Specifically, it involves disruption of amino acid and carbohydrate metabolism, alterations in glycerophospholipid metabolism and disturbances in bile acid metabolism [8–11]. However, only few studies have focused on the relationship between the therapeutic effects of antidepressants and the gut microbiome. Previous studies have suggested that, apart from their direct effects on serotonergic neurons in the gastrointestinal (GI) tract [12], SSRI antidepressants may possess antimicrobial effects that lead to the disruption of the integrity and stability of the gut microbiome [13, 14]. In recent years, the modulatory effects of antidepressants on the gut microbiota have been observed in human and animal models [15–17]. Mice treated with antidepressants (fluoxetine, escitalopram, venlafaxine, duloxetine or desipramine) display changes in the diversity and composition of the gut microbiota, including reduced richness and abundances of *Ruminococcus* and *Adlercreutzia* [15]. Conversely, gut microbes can also influence individual responses to drugs by chemically modifying and altering the bioavailability, bioactivity or toxicity of the drug [18]. Indeed, there is evidence suggesting that specific bacteria can accumulate the antidepressant duloxetine intracellularly, leading to reduced drug availability and potentially impacting its effectiveness. This phenomenon, known as bioaccumulation, can result in altered drug metabolism within the bacteria that have accumulated the drug [19]. However, most of the previous studies have primarily focused on in vitro and in vivo aspects, and there is a relative scarcity of clinical studies specifically conducted on patients with MDD who are undergoing antidepressant treatment. In a particular study with a limited sample size of 30 patients with MDD, the researchers observed that patients whose depressive symptoms improved, as indicated by a Hamilton Depression Rating Scale (HAMD) score greater than 50%, exhibited a significant decline in alpha diversity of the gut microbiome [17]. Many previous studies have primarily focused on examining the changes in single "omics", such as analyzing the gut microbiota composition or investigating gene expression patterns in the brain. However, this narrow focus limits our understanding of the complex interactions between the gut microbiota and the brain. To gain a more comprehensive understanding, it is crucial to conduct systemic research that considers the simultaneous changes across different “omics” aspects. Integrating data from multiple omics can provide a more holistic view of the molecular interactions and pathways involved in the gut-brain axis. Furthermore, the lack of biomarker-based prediction of antidepressant efficacy poses a significant challenge. Identifying reliable biomarkers that can predict individual response to antidepressant treatment is essential for personalized medicine approaches in MDD.

To address this knowledge gap, we performed a longitudinal multi-omics analysis of fecal and plasma samples from 110 patients with MDD in order to investigate the effects of escitalopram (ESC, a SSRI antidepressant) on gut microbiota and the relationship between gut microbiota and ESC efficacy. Firstly, we conducted a comprehensive analysis of longitudinal changes in gut microbial composition, microbial functions, and both fecal and plasma metabolite profiling over a 12-week period of antidepressant treatment. Next, we investigated the role and contribution of the gut microbiota in mediating antidepressant remission. Lastly, we explored the potential of the baseline gut microbiota as a predictive factor for clinical antidepressant remission. By analyzing the initial microbial composition of individuals before treatment, we sought to determine if specific microbial markers or patterns could serve as indicators for the likelihood of achieving remission with antidepressant therapy.

## Results

### Clinical characteristics and data collection

**Fig. 1:**
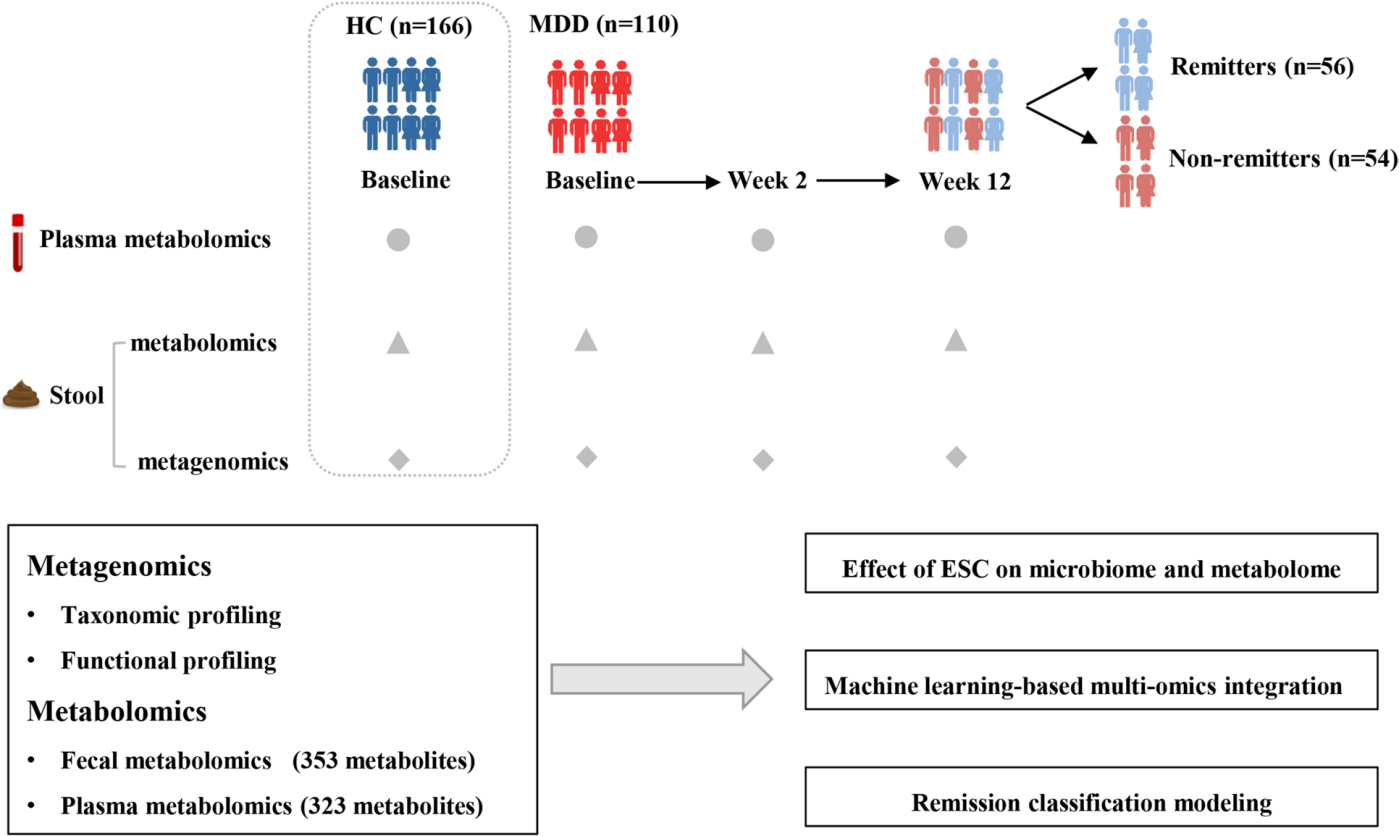
Overview of the study design. The workflow schematic diagram for this study illustrates the details of sample groups and multi-omic data collection. MDD patients were treated with ESC for 12 weeks, and treatment outcomes were evaluated at week 2 (early) and week 12 (endpoint). All patients were divided into remitter (R, HAMD-17 ≤ 7) and non-remitter (NR, HAMD-17 > 7) groups according to the HAMD-17 score at week 12

One hundred and ten MDD patients (41 males and 69 females) who received ESC monotherapy from a large cohort were included in this study [20–22]. At the same time, 166 healthy individuals (68 males and 98 females) were matched as healthy controls (HCs). It is worth noting that a majority of these individuals were selected from our previously published study [11]. The overall workflow of this study is shown in Fig. 1. There were no significant differences in age, sex and BMI between the MDD and HC cohorts (all *P* > 0.05) (details of all individuals are presented in Table S1). After 12 weeks of ESC treatment, the 17-item Hamilton Depression Rating Scale (HAMD-17) scores of MDD patients were significantly reduced (*P* < 0.05). Among them, 56 patients (50.91%) achieved remission (defined as remitters with HAMD-17 ≤ 7) and 54 patients (49.09%) remained in a non-remission state (defined as non-remitters with HAMD-17 > 7). The internationally recognized standard for defining remission is when the score on the Hamilton Depression Rating Scale (HAMD-17) is less than or equal to 7 [4, 23]. The reliability and validity of the HAMD-17 have been tested on the Chinese population [24]. With the exception of education level and baseline HAMD score, there were no significant differences in other baseline characteristics between the remitters (R) and non-remitters (NR) groups (Table S2). Among the recruited subjects, a sub-cohort of 59 patients with MDD (34 R and 25 NR) and 84 HCs had completed the “Diet and Lifestyle Questionnaire”. This questionnaire covers various aspects, including medical history, dietary preferences, smoking, antibiotics and probiotics. No significant differences in these potential confounders were observed between the two populations (Tables S3 and 4).

A total of 285 stool and 321 plasma samples were collected from MDD patients at baseline, week 2 and week 12 after ESC treatment. For HCs, 166 stool and plasma samples were collected at baseline, respectively. All the stool samples were subjected to shotgun metagenomic sequencing and non-targeted metabolomics. Meanwhile, the plasma samples were also subjected to non-targeted metabolomics. In total, 4.86 Tb of metagenomic data were generated in this study, with an average of 10.58 Gb per sample. After removing low-quality reads and host DNA, there was an average of 93.5% high-quality sequences remaining, with a range of 57.6% to 96.2%*.* Finally, 353 fecal metabolites and 323 plasma metabolites were obtained.

### ESC treatment ameliorates the dysregulation of plasma metabolites in MDD

**Fig. 2:**
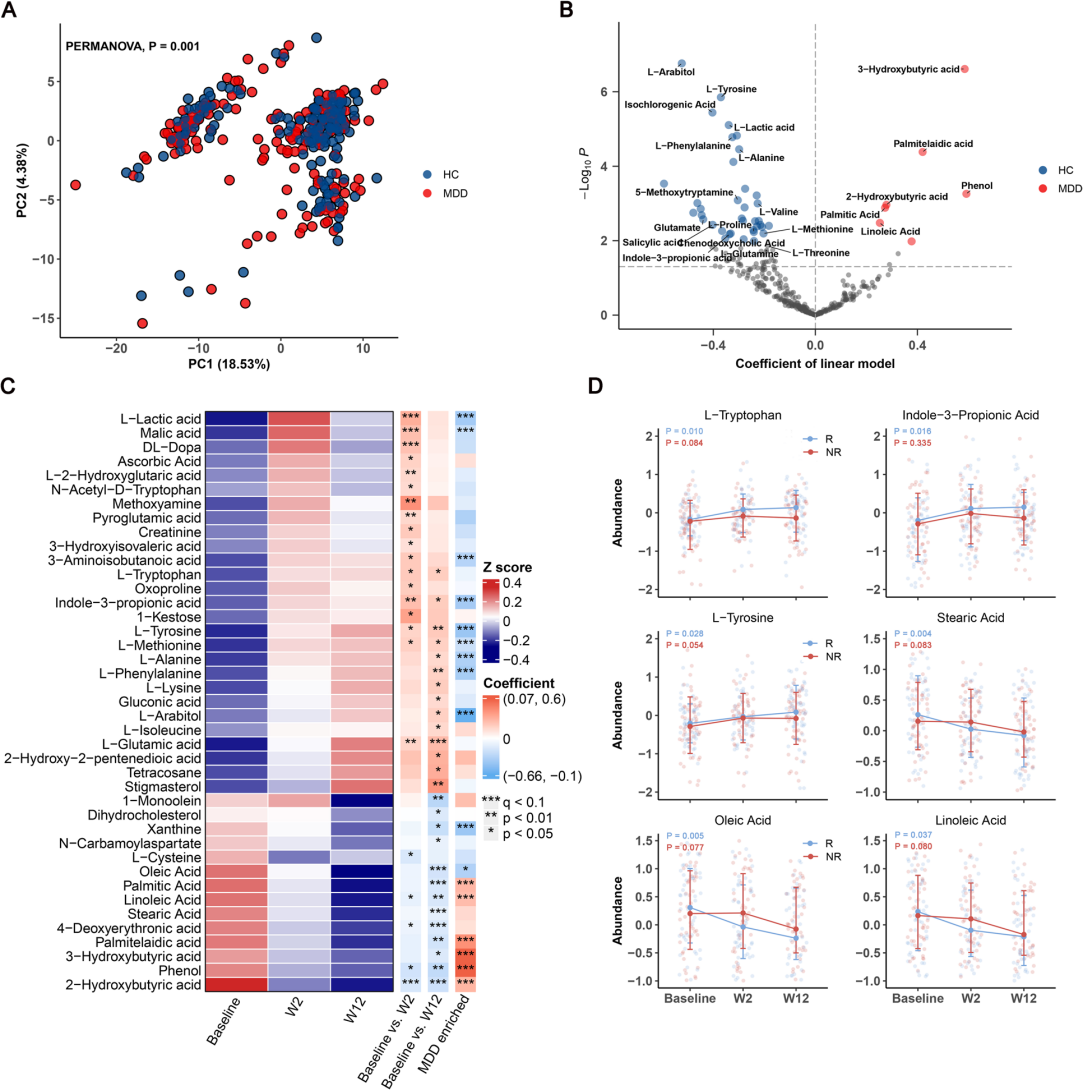
The dysregulated plasma metabolome of MDD patients, and the effect of ESC treatment on plasma metabolites. **a** Principal component analysis (PCA) based on plasma metabolomic profiles revealed a significant difference between the plasma metabolic signatures of MDD patients and those of HCs (PERMANOVA, Euclidean distance, *P* = 0.001). **b** Volcano plot of regression coefficients for the differences in metabolite levels between MDD patients and HCs. Multivariate regression models were used to explore the associations between plasma metabolites and disease status, statistical significance was determined by q value < 0.1. Compared with HCs, the MDD group was characterized by 7 up-regulated metabolites and 37 down-regulated metabolites, these metabolites were mainly involved in organic acid and amino acid. **c** Heatmap showing the average abundance of 41 plasma metabolites, which were significantly changed at week 2 or week 12 after ESC intervention, as determined by linear mixed models (LMM). Heatmap row annotation showing the colored coefficients derived from LMMs (Baseline vs. W2 and Baseline vs. W12) and multivariate regression models (MDD vs HC). The coefficients have different colors of blue (down-regulated or enriched in HC) and red (up-regulated or enriched in MDD). Significant differences are indicated by * *p* < 0.05, ** *p* < 0.01, *** q < 0.1. **d** Longitudinal changes in 6 plasma metabolites (from c), which exhibited differential trends between the R and NR groups. The *P* values were obtained from LMMs applied to grouped data of R and NR

The overall plasma metabolic signatures of MDD patients were significantly different from those of HCs, as illustrated by PCA (PERMANOVA, Euclidean distance, *p* = 0.001, Fig. 2A). Compared to HC group, MDD group showed enrichment in 7 metabolites and depletion in 37 metabolites (q < 0.1; Fig. 2B). MDD patients were characterized by higher levels of organic acid metabolites (most of these belong to fatty acid), such as 3-hydroxybutyric acid, palmitelaidic acid, 2-hydroxybutyric acid, palmitic acid, and linoleic acid, as well as lower levels of amino acid metabolites, including L-tyrosine, L-phenylalanine, L-alanine, L-valine, L-proline, L-methionine and glutamate.

PERMANOVA was simultaneously employed to analyze the changes in plasma metabolites after ESC treatment. In this scenario, PERMANOVA tests were implemented using a mixed-model design, with subject ID included as a blocking factor ("strata") to control for repeated sampling. It was observed that significant changes in overall metabolic characteristics occurred at week 12, but not at week 2 (Euclidean distance, week 2, *p* = 0.31; week 12, *p* = 0.032; as illustrated by PCA in Figure S1A). Next, to clarify the relationship between plasma metabolome and drug efficacy, PC1 (11.1% variation), PC2 (8.24% variation) and PC3 (5.11% variation) were obtained from the principal component analysis (PCA) data of plasma metabolome profiling. Then, the relationship between HAMD-17 scores and different principal components was established using linear mixed model (LMM). The model demonstrated that HAMD-17 score was significantly negatively correlated with PC3 (coefficient β = -0.2, *p* = 0.0101). These findings suggest that the changes in plasma metabolome are associated with depressive symptoms.

To examine the alterations in plasma metabolic components after ESC treatment, the linear mixed models (LMMs) were carried out, which incorporated visit week, age, sex, education, BMI, and month of disease duration as fixed effects, and subject ID as random effect. Using a significant threshold of q value < 0.1, we identified changes in 4 and 8 plasma metabolites after 2 and 12 weeks of treatment, respectively (33 and 39 if using *p* < 0.05). As shown in Fig. 2C, various amino acid metabolites were upregulated, including L-tyrosine, L-methionine, L-phenylalanine, L-alanine and L-tryptophan. An important observation to highlight is that L-tryptophan serves as the precursor for the neurotransmitter 5-hydroxytryptamine (5-HT), commonly known as serotonin. L-tryptophan has the ability to pass through the blood–brain barrier, and plasma levels of tryptophan are generally regarded as a reliable indicator of central 5-HT activity [25]. Interestingly, despite the ESC treatment, we did not observe an increase in serum 5-HT levels. However, our findings revealed that another gut microbiota-derived metabolite of tryptophan [26, 27], indole-3-propionic acid (I3PA), was markedly upregulated. I3PA has recently been reported to promote nerve regeneration and repair [28]. In contrast, organic acids (most of these belong to fatty acid) were significantly down-regulated, including stearic acid, oleic acid, linoleic acid, 2-hydroxybutyric acid, palmitelaidic acid, 4-deoxyerythronic acid, 3-hydroxybutyric acid, phenol and palmitic acid. Fisher exact test showed that the up-regulated metabolites were enriched in HC-enriched metabolite sets, while the down-regulated metabolites were enriched in MDD-enriched metabolite sets (*p* = 6.73e-05). Of note, 6 up-regulated and 2 down-regulated metabolites overlapped with HC-enriched metabolites, while 0 up-regulated and 6 down-regulated metabolites overlapped with MDD-enriched metabolites. Taken together, these findings imply that ESC treatment ameliorates the dysregulation of plasma metabolites in MDD patients by up-regulating amino acid metabolism and down-regulating fatty acid metabolism.

Furthermore, we examined the differential effects of ESC treatment on the blood metabolome in R and NR groups separately using LMMs. After controlling baseline plasma 5-HT levels and confounding effects, we found that the 5-HT level in R group was higher than that in NR group after ESC treatment (barely significant, *p* = 0.077; Figure S1B). Interestingly, the “good” metabolites (HC-enriched metabolites, such as L-tyrosine, L-tryptophan, and I3PA) and “bad” metabolites (MDD-enriched metabolites, such as stearic acid, oleic acid, and linoleic acid) were up-regulated and down-regulated only in R group (Fig. 2D). In addition, primary bile acids (cholic acid and chenodeoxycholic acid), which can be metabolized by gut microbiota and play an important role in regulating the gut-brain axis [29], also exhibited different trends between subgroups (Figure S1C). These results indicated that the changes in plasma metabolites after ESC treatment were different between the R and NR groups, which may be attributed to the treatment outcome of ESC. It is worth highlighting that some of the metabolites that showed alterations in response to the treatment, including tryptophan and I3PA, are metabolites associated with the gut microbiota. This observation strongly suggests the involvement of the gut microbiota in the mechanism of action of ESC.

### ESC treatment exerts an inhibitory effect on gut microbiota

**Fig. 3:**
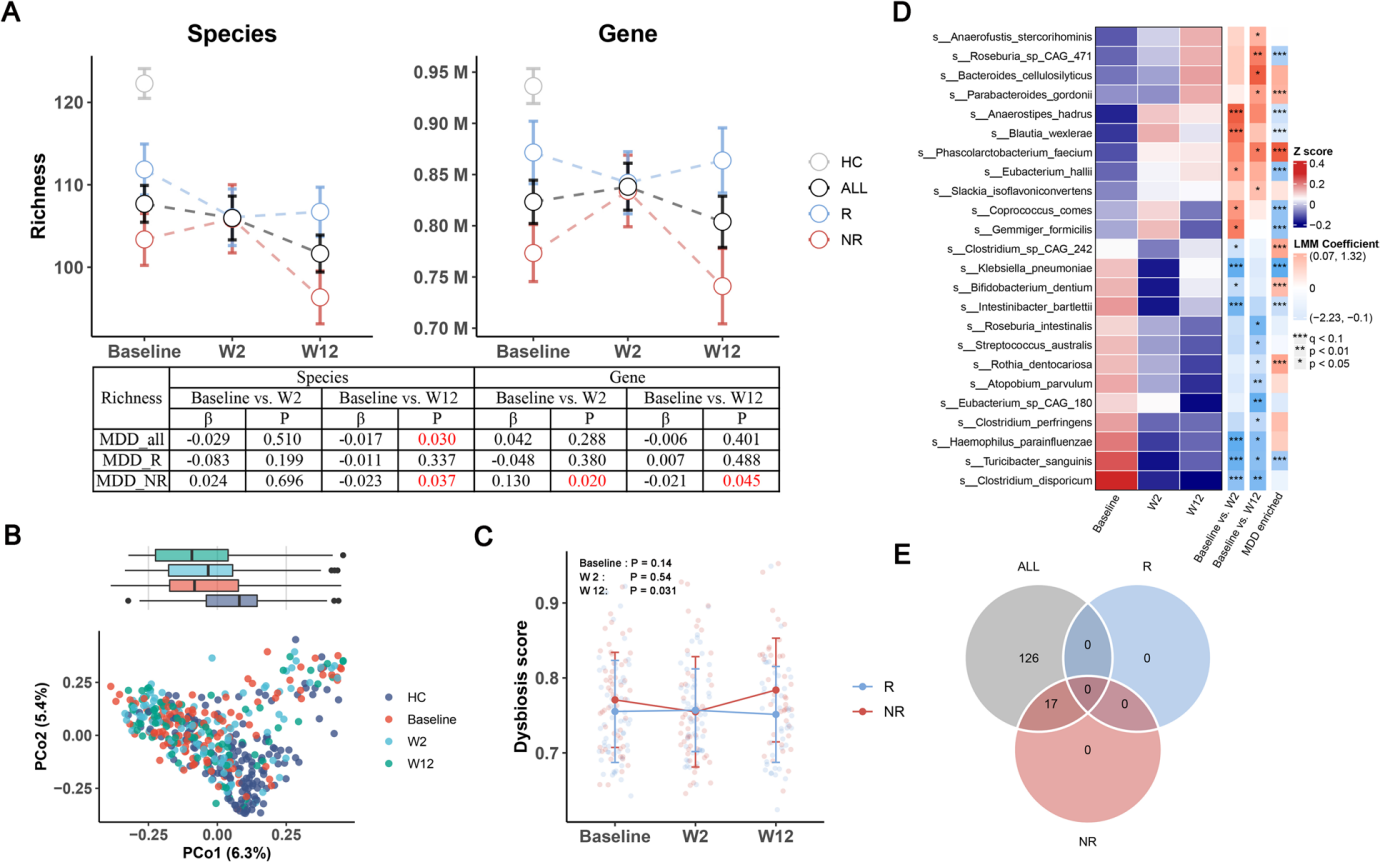
Effects of ESC treatment on the gut microbiome. **a** Changes in microbial richness at species and gene level from baseline to week 12 in ALL, R and NR groups, with HC as reference. ALL included the samples from both R and NR groups. The table presents detailed results from longitudinal comparisons using LMM, with visit week coefficients and *P* values. **b** Principal coordinate analysis (PCoA) plots based on the metagenomic profiles of different groups. **c** Changes in dysbiosis score (DS) from baseline to week 12 in the R and NR groups. NR was significantly higher than R at week 12 (*P* = 0.031). **d** Heatmap showing the average abundance of 24 species, which were significantly changed at week 2 or week 12 after ESC intervention, as determined by linear mixed models. Heatmap row annotation showing the colored coefficients derived from LMMs (Baseline vs. W2 and Baseline vs. W12) and multivariate regression models (MDD vs HC). The coefficients have different colors of blue (inhibited or enriched in HC) and red (promoted or enriched in MDD). Significantt differences are indicated by * *p* < 0.05, ** *p* < 0.01, *** q < 0.1. **e** Venn diagram of significantly downregulated metaCyc pathways in ALL (q < 0.25), R (q < 0.25) and NR (q < 0.25) groups after ESC intervention

Next, we focused on the effects of ESC on the gut microbiota. As shown in Fig. 3A, ESC treatment significantly reduced the richness at the species level, but not at the gene level. Upon subgroup analysis, we observed that the decrease in microbial richness was specifically evident in NR group from baseline to week 12. However, it is important to note that at the gene level, there was a temporary increase in richness observed from baseline to week 2. In R group, there was no significant change in richness at the species or gene level. Moreover, the richness of R group was significantly higher than that of NR group at both baseline and week 12, but consistently lower than that of HC group. Similarly to the richness analysis, we observed a similar trend in the Shannon index (Figure S2A). These results serve as a reminder that the effect of ESC on the gut microbiota varied between the R and NR groups, as well as between the short-term (baseline to week 2) and long-term (baseline to week 12) treatment periods.

PERMANOVA results showed that the gut microbial composition changed significantly at week 12 (constrained PERMANOVA, R^2^ = 0.0046, *p* = 0.034), but not at week 2 (Fig. 3B). Our observations indicated that even at week 12, there remained a noticeable distinction in microbial composition between MDD patients and HCs at week 12 (Bray–Curtis distance, R^2^ = 0.027, *p* = 0.00033). This suggests that ESC treatment did not effectively shift the gut microbiome of MDD patients towards a healthier or more similar state to that of HCs. However, HAMD-17 score was negatively correlated with PCoA1 (LMM, coefficient β = -3.39, *p* = 0.05), which indicted a significant association between the remission of depressive symptoms and changes in the gut microbiota. To further explore whether the changes in the gut microbiota of MDD patients were directed towards a healthier state, we estimated a dysbiosis score (DS) for each MDD sample, using HC samples as a reference, and calculated the median value of Bray–Curtis distance between the MDD and HC cohorts [30]. Our findings revealed a significant negative correlation between the DS and species richness (Figure S2B). Overall, DS did not alter significantly from baseline to week 12 (*p* = 0.276). However, DS was higher in NR group than in R group at baseline and week 12, with a significant difference at week 12 (*p* = 0.031; Fig. 3C). In light of the findings, ESC did not improve the overall gut microbial status, but NR group had a more disturbed gut microbiota compared to R group.

At the individual taxa level, our analysis revealed significant changes in specific species following ESC treatment. Specifically, from baseline to week 2, we observed the promotion of five species and the inhibition of seven species. Similarly, from baseline to week 12, ESC treatment resulted in the promotion of six species and the inhibition of nine species (Fig. 3D). In contrast to the previous findings regarding plasma metabolites, we did not observe any enrichment of the promoted or inhibited species in both HC and MDD cohorts. Among the species that showed significant alterations following ESC treatment, it was observed that several of them belonged to spore-forming bacteria, including *Clostridium disporicum, Turicibacter sanguinis, Eubacterium hallii, Coprococcus comes* and *Clostridium perfringens*. A previous study reported that spore-forming bacteria, such as *T. sanguinis* and *Clostridiaceae*, could interact with enterocytes to increase 5-HT production, while SSRIs could inhibit SERT on the surface of *T. sanguinis* and affect its absorption of 5-HT, thereby increasing the concentrations of 5-HT in the intestine [31]. In our study, *T. sanguinis* (q = 0.057) and *Clostridiaceae* (q = 0.12) were also significantly reduced from baseline to week 2, with *Clostridiaceae* only showing a decrease in R group (q = 0.078) rather than NR group. Given the relationship between spore-forming bacteria and SSRIs, we further investigated the changes in sporulation genes after ESC intervention. In view of this, we obtained the sequences of 66 sporulation signature genes from a previous study [32], and 51 of the 66 genes could be hit by our non-redundant gene catalogue using BLAST. The abundance of the 51 sporulation genes was obtained by summing up the abundance of each hit gene. It was found that sporulation genes were significantly down-regulated and this down-regulation pattern occurred only in the R group (Figure S2C). After performing the taxonomic assignment, the majority of the sporulation genes were annotated to the order Clostridiales, accounting for approximately 74.74% of the assignments. Another noteworthy observation in our study was the significant increase in the Firmicutes phylum, which is a phylum known to encompass several members of spore-forming bacteria [33], from baseline to week 2 (q = 0.0089), particularly in the NR group (q = 0.039; Figure S2D). In addition to the observed increase in Firmicutes from baseline to week 2, our analysis revealed a positive correlation between the change in Firmicutes abundance and the change in species richness (Figure S2E). Furthermore, considering the transient increase in richness specifically from baseline to week 2 in the NR group, this result implies that the increase in richness may be related to the concurrent increase in Firmicutes abundance during that period.

Among the bacterial species affected by ESC treatment, we also found some species that changed differentially between the R and NR groups (Figure S2F), including *Intestinibacter bartlettii*, *E. hallii*, *Roseburia intestinalis*, and *Phascolarctobacterium faecium*. *I. bartlettii,* a bacterium that is known to have potentially harmful effects [34], exhibited a greater decrease in the R group following ESC treatment. Notably, at week 12, the NR group showed a higher abundance of *I. bartlettii* compared to the R group. On the other hand, *E. hallii* showed an increase in the R group and a decrease in the NR group. At week 12, the R group had a higher abundance of *E. hallii* compared to the NR group, although the difference approached significance (*p* = 0.067). A noteworthy finding in our study is the depletion of *E. hallii* in MDD patients compared to HCs. This observation is consistent with our previous study [11], further reinforcing the association between the reduced abundance of *E. hallii* and MDD risk. *E. hallii*, as a key species within the intestinal trophic chain, has the potential to strongly influence the metabolic balance as well as the gut microbiota/host homeostasis through the production of different short chain fatty acids (SCFAs) [35]. Furthermore, our analysis revealed that *R. intestinalis* (*p* = 0.087) and *P. faecium* (*p* = 0.058), both known as SCFA-producing bacteria, exhibited a higher overall abundance in R group than in NR group [36, 37].

In terms of microbial functions, the changes in metabolic pathways were analyzed using MaAsLin2, with the subject as a random effect. After ESC treatment, 145 pathways were significantly altered in all samples, of which 143 pathways were down-regulated (q < 0.25). Further subgroup analysis showed that 17 pathways were down-regulated in NR group, whereas no pathway was altered in R group (using a significance threshold of q < 0.25, Fig. 3E). Considering the known antimicrobial properties of SSRIs [31], we also determined the impact of ESC on antimicrobial resistance (AMR) genes. our findings revealed an overall up-regulated trend of AMR genes following ESC treatment. Specifically, out of the 15 ESC-altered genes analyzed, 11 of them showed upregulation, using a significance cutoff of *p* < 0.05 (Table S5). In contrast to the overall up-regulated trend of AMR genes observed after ESC treatment, our analysis revealed that only a specific AMR gene family, namely the major facilitator superfamily (MFS) antibiotic efflux pump, showed significant up-regulation in R group when using a significance threshold of q < 0.25. It has been reported that antidepressants could induce resistance to multiple antibiotics by inducing the expression of efflux pumps [38]. Taken together, these findings suggested indicate that ESC treatment exerts an inhibitory effect on microbial functions, particularly in NR group, whereas R group showed greater resistance to ESC perturbation, which may be more conducive to drug efficacy.

As the fecal metabolome can provide a functional readout of the gut microbiome, GC–MS-based metabolomic analysis was simultaneously performed to compare the microbial metabolic signatures after ESC treatment. Although there was an obvious effect of ESC on microbial composition and functions, we did not observe significant changes in fecal metabolites after FDR correction (using a significance threshold of q < 0.1). When using *p* < 0.05 as the significant threshold, 22 fecal metabolites significantly altered at week 12, but all had q > 0.5 after FDR correction. In the NR group, we observed only 9 significant changes in fecal metabolites from baseline to W2 (q < 0.25, Table S6). These findings were consistent with a significant change in gene diversity from baseline to W2 only in the NR group (Fig. 3A). However, this consistency disappeared from baseline to W12, possibly due to metabolomic technical bias or biological confounding factors that we were unable to account for or capture. Therefore, we redirected our focus towards exploring the association between the plasma metabolome and the gut microbiome in the subsequent part of our study.

### Integration network of plasma metabolome and gut microbiome

**Fig. 4:**
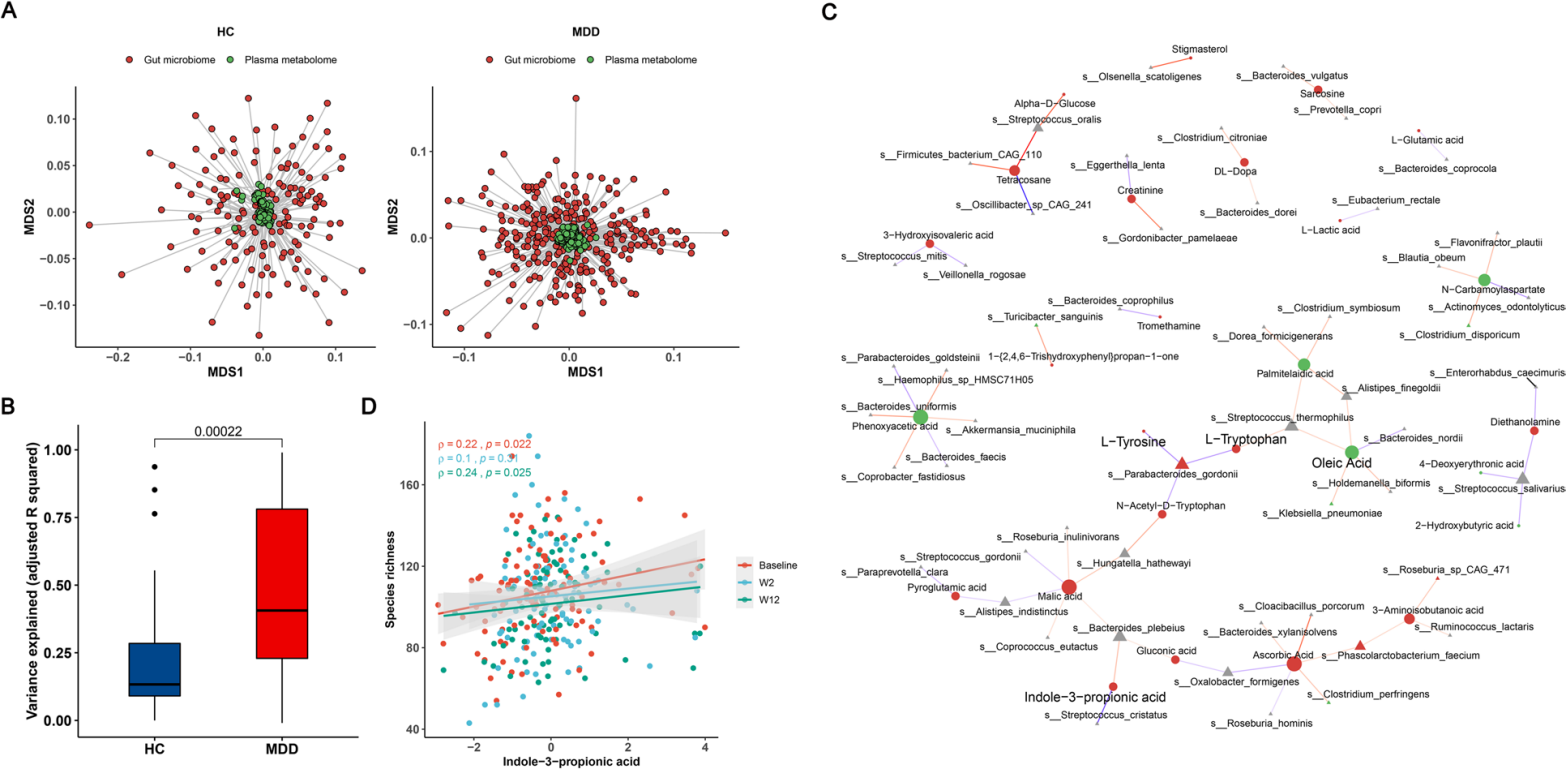
Association between plasma metabolome and gut microbiome. **a** Multiple dimensional scaling (MDS) plot of procrustes analysis showing overall association between plasma metabolome and gut microbiome in MDD and HC cohorts, with individual samples being connected by a line. Euclidean distance was used for plasma metabolome (green circles), while Bray–Curtis distance for gut microbiome data (red circles), and the procrustes m^2^ statistic results were labeled. **b** Compared to HCs, the variance of plasmas metabolite explained by gut microbiota was significantly higher in MDD patients. The extent of variance explained was quantified using the r.^2^ statistic derived from a lasso model. **c** Association network between plasma metabolites and microbial species generated from glmmLasso models. The analysis presented only those metabolites that showed significant alterations in response to ESC treatment. Node size indicates degree, different shapes represent metabolites (circles) and microbes (triangles), different colors of nodes represent up-regulated (red) and down-regulated (green) after ESC intervention, negative correlations are shown in blue, while positive correlations are shown in red. **d** Scatter plot revealing the spearman's correlation between the species richness and indole-3-propionic acid (I3PA) at three visit weeks. The significant positive correlations were only observed at baseline and week 12

The aforementioned findings suggest that ESC treatment influenced both the gut microbiome and the plasma metabolome. These results align with previous findings indicating that the gut microbiota can play a role in modulating individual plasma metabolite profiles, with the extent of influence varying depending on the specific disease or condition [39, 40]. Thus, we continued to explore the association between the two sets of omics data. We first performed a Procrustes analysis using sample matched omics data from both HC and MDD cohorts, respectively. The results revealed a weak correspondence between the plasma metabolome and gut microbiome in MDD patients (Monte Carlo *P* value = 0.013) and HCs (Monte Carlo *P* value = 0.092; Fig. 4A). As anticipated, we observed a stronger correlation between the fecal metabolome and gut microbiome in both MDD (Monte Carlo *P* value = 0.0445) and HC (Monte Carlo *P* value = 5e-04) cohorts (Figure S3A). Given the lack of significant overall association between the plasma metabolome and gut microbiome, we speculate the association is more likely to occur at the individual level between the metabolite and gut microbiota, rather than an overall inter-omics association.

To address this hypothesis, we employed a machine learning framework as proposed in a previous study [41]. This framework implements lasso penalized regression to characterize potential biologically meaningful associations between genes and gut microbiota. By applying this approach, we aimed to explore specific relationships between plasma metabolites and gut microbiota that could contribute to our understanding of the underlying biological mechanisms of depressive symptoms. The lasso regression uses shrinkage to perform feature selection, retaining only a few taxa associated with a plasma metabolite. Through this approach, we discovered 169 significant plasma metabolite-taxa associations in the HC group, while 680 associations in the MDD group (q < 0.1; Figure S3B). These represent associations between 40 plasma metabolites and 119 gut microbes in HCs (Table S7), and between 60 metabolites and 219 gut microbes in MDD patients (Table S8). An interesting observation was made regarding the overlap of associations between plasma metabolites and gut microbiota in the two populations. Specifically, there were no shared associations between the HC and MDD groups, with only six metabolites found to be associated with microbial taxa in both populations (Figure S3B). This lack of overlap suggests that the potential biological associations between plasma metabolites and gut microbiota differ between the two populations. However, given that the lasso model is sensitive to small variations of the predictor variable (gut microbe), the difference between the two populations may be partly attributed to the selection method [41]. Additionally, our analysis revealed a significant difference in the variance of plasma metabolites explained by gut microbiota between the MDD and HC groups, with a higher variance explained in the MDD population (Fig. 4B). These results suggest that gut microbes have a stronger impact on plasma metabolites in individuals with MDD compared to healthy controls. Overall, MDD-specific patterns are detected in the associations between the plasma metabolite and gut microbiota.

Next, to further establish the association between individual plasma metabolites and gut microbiota, glmmLasso models were constructed using all MDD samples. For each metabolite, the glmmLasso model was fitted to select the potentially associated bacterial species, and these selected features were further fitted to the LMM model to assess its significance levels (see Methods). A total of 255 significant associations were found between 109 metabolites and 139 species after FDR correction (q < 0.1, Table S9). As shown in Fig. 4C and Table S9, 19 metabolites altered by ESC treatment were associated with bacterial species, and the number of up-regulated metabolites was much higher than that of down-regulated metabolites. For example, L-tryptophan was associated with *Parabacteroides goldsteinii* and *Streptococcus thermophilus*. The latter belongs to tryptophan-metabolizing bacteria, which contains tryptophanase that can maintain the growth and survival of bacteria through tryptophan metabolism, and the metabolites produced by these bacteria can regulate microbial diversity and confer benefits to the host [42–44]. In our analysis, we found that I3PA was associated with *Bacteroides plebeius* and *Streptococcus cristatus*. Furthermore, we observed that I3PA was significantly positively correlated with species richness (Fig. 4D). Taken together, the above findings suggest a potential correlation between altered plasma metabolites and gut microbes after ESC treatment, in which amino acid metabolism and tryptophan derivatives may play an important role.

### Baseline microbiome characteristics of different remission groups

**Fig. 5:**
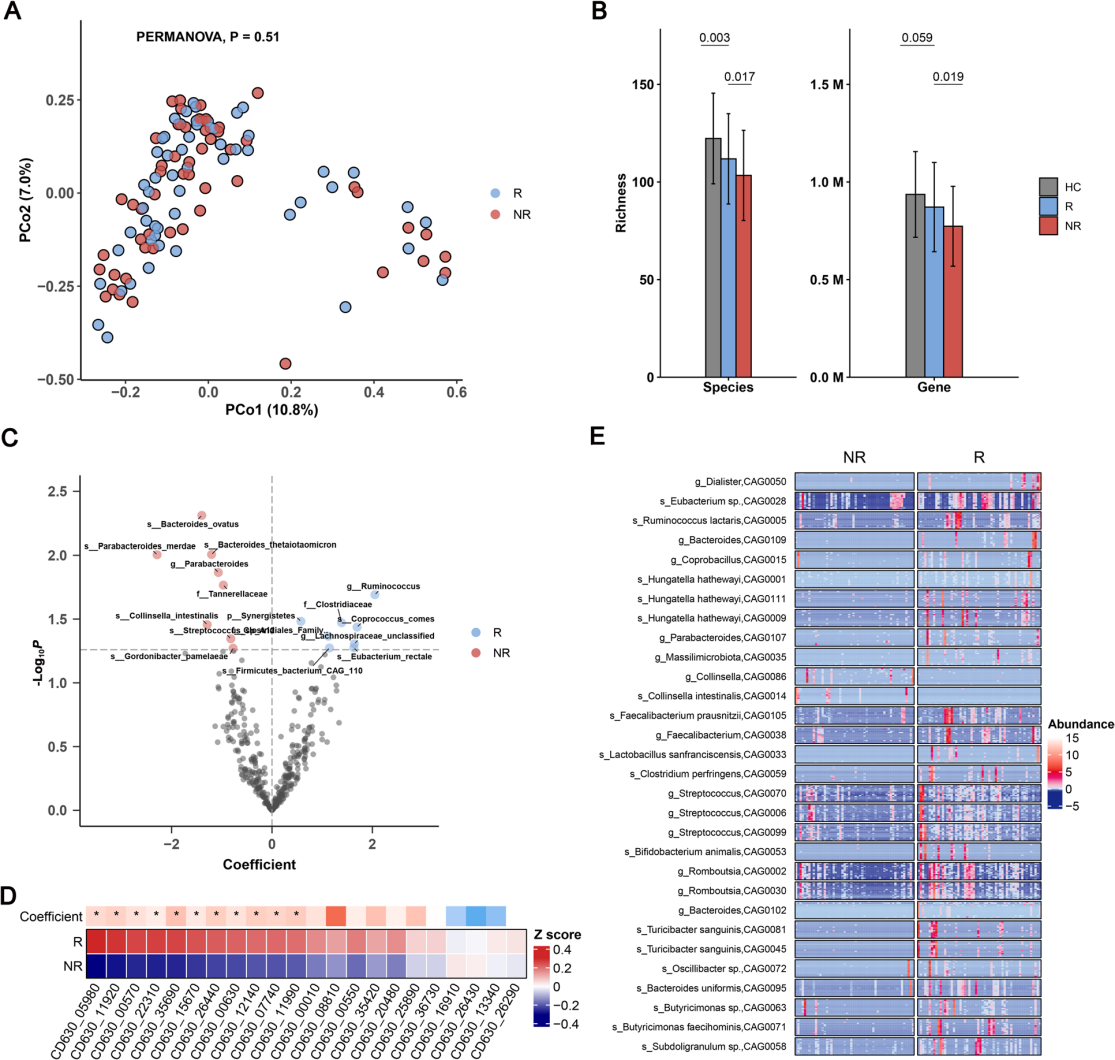
Microbiome comparison between the R and NR groups at baseline. **a** PCoA plot showing no significant difference in microbial composition between the R and NR groups using Bray–Curtis dissimilarities (PERMANOVA *P* = 0.5). **b** Richness difference of microbial species (left) and gene (right) between the R and NR groups at baseline, with HC as reference. **c** Volcano plot of regression coefficients for gut microbial species in remission group. Multivariate regression models were used to explore the associations between gut microbiota and clinical remission. A *p*-value of < 0.05 was considered statistically significant. In R group, the higher abundance species are shown in blue dots, while lower abundance species are shown in red dots. **d** Heatmap showing the average abundance of 22 sporulation genes, of which 11 were enriched in R group, as determined by multivariate regression models. Heatmap row annotation showing the colored coefficients derived from MLR models (R vs NR). The coefficients have different colors of blue (enriched in NR) and red (enriched in R). Significant differences are indicated by * *p* < 0.05. **e** Heatmap of co-abundance groups (CAGs) of differentially abundant genes between the R and NR groups at baseline. Gene abundance is indicated by color gradient (red indicates the highest abundance, white indicates zero, and blue indicates the lowest abundance)

One of the primary objectives of our study was to investigate the predictive potential of gut microbiota for antidepressant efficacy. In line with this objective, we aimed to compare the microbiome characteristics between the R and NR groups at baseline. Although PERMANOVA analysis showed that there was no significant difference in microbial composition between the two subgroups at baseline (*p* = 0.51; Fig. 5A), the microbial richness of R group was significantly higher than that of NR group at both species (*p* = 0.017) and gene (*p* = 0.019) levels (Fig. 5B). Furthermore, species richness was negatively correlated with the HAMD-17 at baseline (Figure S4A). The Shannon index also exhibited a similar trend (*p* = 0.082, *p* = 0.002), although there was no significance at the species level (Figure S4B). Indeed, diversity within the gut microbiota has been reported to play a positive role in promoting microbial resilience and enhancing its stability [45]. To further estimate the resilience of the microbial community, we constructed microbial interaction networks for both R and NR groups using sparCC, and found that the community structure and bacterial networks in R group were more complex and better organized than those in NR group (Figure S4C). Then, we compared the resilience of the two networks by randomly removing nodes to simulate ‘ecological attacks’ to the network [46]. Natural connectivity was used to assess the robustness of the remaining networks [47]. Although under ‘attacks’, natural connectivity was consistently higher in the R network compared to the NR network (Figure S4D). These results supported a more resilient bacterial community in R group. In addition, we observed that several spore-forming taxa were more abundant in R group than in NR group, including *Clostridiaceae*, *Eubacterium rectale* and *C. comes* (*P* < 0.05; Fig. 5C). Spore-forming bacteria can resist external environmental stimuli, promote gut microbiota reconstruction, and restore intestinal homeostasis [48]. It is intriguing to note that *E. rectale*, a core gut commensal species, consistently showed higher abundance in R group compared to NR group at three visit weeks, and its abundance was positively correlated with species richness at baseline and week 12 (Figure S4E&F). As a strict anaerobe, *E. rectale* can promote host gut health by producing butyrate and other SCFAs from indigestible fibers [49], and has recently been identified as psychobiotics to maintain and improve mental health [50]. Similar to the situation of spore-forming bacteria, the abundance of most sporulation genes (11 out of 22 sporulation genes that occur in at least in 20% of baseline samples) was higher in R group than in NR group (q < 0.1, Fig. 5D). In addition, 18 out of 22 sporulation genes showed a significant positive correlation with the species richness (Table S10). Thus, we speculate that microbial sporulation may contribute the resistance of gut microbiota to ESC-induced perturbation.

To better understand the microbial differences between the R and NR groups at baseline, we compared the abundances of non-redundant genes between the two subgroups using the Wilcox test, and obtained a total of 32,231 differential genes (*p* < 0.01). The differential genes were clustered into co-abundance groups (CAGs) using the canopy clustering algorithm and the CAGs were then subjected to taxonomic annotation. As shown in Fig. 5E, the majority of the differential genes were annotated to Clostridales (78.40%). Compared to NR group, the CAG-annotated species, butyrate-producing bacteria including *Ruminococcus lactaris*, *Faecaecalibacterium prausnitzii*, *E. rectale* and *Romboutsia* were enriched in R group. Butyrate exhibits a positive impact on the balance of the gastrointestinal tract by inhibiting inflammation and carcinogenesis, strengthening various components of the colonic defence barrier, and reducing oxidative stress [51, 52]. Besides, the beneficial bacteria annotated by CAG, such as *Bifidobacterium animalis* and *Lactobacillus sanfranciscens*, were also enriched in R group. Our results support the notion that the overall gut microbiome status of R group may be better than that of NR group at baseline.

### Sporulation genes as a predictor of MDD remission in response to ESC

To explore the predictive potential of the gut microbiota at baseline for treatment remission in MDD patients, we constructed prediction models using three different feature sets (taxa, diversity and sporulation gene) derived from the baseline samples.

**Fig. 6:**
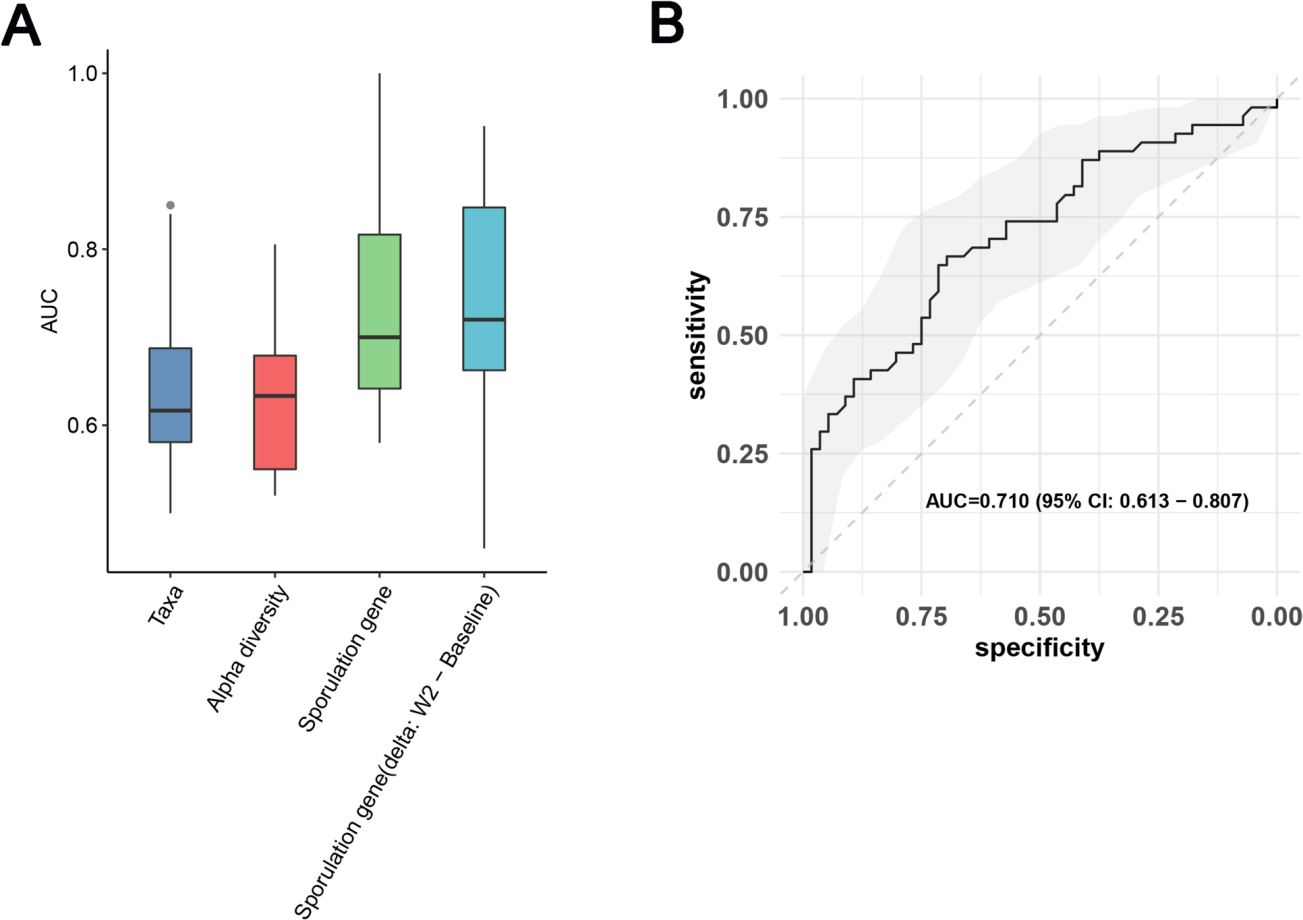
Predictive values of gut microbiome features at baseline on the clinical remission of MDD patients. **a** AUC boxplot of 10 outer loop testing results for each feature set, random forest was used to train data, mean AUC ranging from 0.56 to 0.73 (Taxa: AUC = 0.65; Alpha diversity: AUC = 0.63; Sporulation gene: AUC = 0.736; Sporulation gene delta between W2 and baseline: AUC = 0.734). **b** Concatenated ROC curve of 10 outer loop testing results using RF model based on sporulation genes

To explore the predictive potential of the gut microbiota at baseline for treatment remission in MDD patients, we constructed prediction models using three different feature sets (taxa, diversity and sporulation gene) derived from the baseline samples.To ensure an unbiased evaluation of the model performance, we employed nested cross-validation in this study (Figure S5A, see Methods). Nested cross-validation is a technique that incorporates both model selection and hyperparameter optimizationprocedures within the evaluation process. Model performance was assessed using the area under the curve (AUC) of the receiver operating characteristic (ROC). In our modelling results, microbial taxa showed a low performance (mean AUC = 0.61; Fig. 6A). However, the highest mean prediction performance was achieved by the sporulation gene model (mean AUC = 0.736) in combination with a ten outer loop model (AUC = 0.710, ROC plotted by merging all outer loop test results; Fig. 6B). Considering the potential influence of ESC on sporulation genes, we also predicted the clinical remission of MDD patients based on the changes in sporulation genes from baseline to week 2. The results demonstrated that the mean AUC and joint AUC were 0.734 and 0.701, respectively, indicating a moderate to good predictive performance for the model (Figure S5B).

## Discussion

In this study, we present a human research endeavor aimed at exploring the effects of antidepressants on the gut microbiota and human metabolism, as well as investigating the potential involvement of the gut microbiota in antidepressant efficacy. To achieve this, we collected multi-omics data from a longitudinal cohort comprising 110 individuals. Our findings revealed that antidepressant treatment had a positive impact on the blood metabolic status of patients diagnosed with MDD. Moreover, we observed that antidepressant use led to a reduction in both microbial diversity and function within the gut microbiota. Furthermore, our study uncovered a significant finding that the characteristics of the baseline gut microbiota can serve as predictors of antidepressant efficacy. This discovery holds great importance as it provides a foundation for comprehending the role of the overall gut ecosystem in the effectiveness of antidepressants. Identifying these predictive markers within the gut microbiota opens up new possibilities for personalized treatment approaches for patients with MDD.

Consistent with the main findings of a previous meta-analysis [53], our results demonstrated that patients with MDD exhibited distinct blood metabolic signatures. Amino acid metabolism and lipid metabolism were obviously perturbed in the peripheral blood of MDD patients, and ESC treatment partially reversed the metabolic dysregulation by up-regulating several amino acids and down-regulating fatty acids. Among the upregulated amino acids identified in the study, L-tyrosine and L-phenylalanine are of particular interest. Both of these amino acids serve as precursors for the synthesis of catecholamine neurotransmitters in the brain. The use of L-tyrosine as a treatment for depression has been investigated in a previous study [54]. Contrary to the findings of a previous meta-analysis [55], our study revealed different results regarding the levels of L-tryptophan in patients with MDD. Specifically, we did not observe lower levels of L-tryptophan in MDD patients compared to controls. and its level was increased after antidepressant exposure. L-tryptophan is a precursor of serotonin, and has been negatively correlated with depression severity in other studies [55]. L-tryptophan is considered an important biomarker for depression and has been investigated as a potential predictor of treatment response to antidepressants. L-tryptophan can also be used to represent the serotonin levels in the brain [25]. Although some studies have shown that the peripheral blood level of 5-HT decreases after treatment with antidepressants [56], our results do not support this conclusion. In fact, our study indicates a nearly significant increase in 5-HT levels among patients who achieved remission compared to those who did not respond as well to the medication. However, a recent research suggests that depression may not be associated with low serotonin levels [57]. To establish a more comprehensive understanding of the impact of ESC on serum 5-HT levels, further larger-scale studies with a diverse participant population are warranted. Our results showed that lipid metabolism dysfunction might play an important role during MDD. Fatty acids may promote depression through multiple pathways, including biological stress and inflammation [58, 59]. Overall, ESC treatment improves depressive symptoms by altering neurotransmitters and relieving depression-associated lipid metabolism pathways.

Among the metabolites altered by antidepressants, I3PA is a derivative of tryptophan that is metabolized by gut microbiota. I3PA levels were significantly depleted in patients with MDD but significantly increased after ESC intervention. These results are consistent with previous studies that have also observed similar changes in I3PA levels following antidepressant treatment [56]. I3PA is an aryl hydrocarbon receptor (AHR) agonist that can activate AHR transcriptional activity and regulate gut and brain inflammation [60, 61]. A recent study found that the gut microbiota-produced IPA could protect the microglia from inflammation, thus promoting neural regeneration and repair [62]. These findings imply that the gut microbiota may have a significant role to play in the mechanism behind the improvement of depression through antidepressant treatments.

In this study, we observed that ESC had an inhibitory effect on the gut microbiota, as evidenced by a decrease in microbial richness and a disruption in most microbial functions. This finding is consistent with a previous in vitro study on the interaction between antidepressants and bacteria showing that ESC has some antibiotic properties [63]. Other studies on antidepressants have also demonstrated a reduction in the diversity of the gut microbiota [15, 17]. However, the results of this study are not consistent with a previous study conducted by Xie et al. [16], which showed an increase in microbial diversity in mice after treatment with ESC. Indeed, the discrepancy between the findings of the two studies could be attributed to several factors, including the diversity index used in the analysis or differences in experimental subjects. To gain a better understanding of these discrepancies, further investigation is necessary. McGovern et al. reviewed the antibacterial properties of SSRIs, and found that different SSRIs can have varying effects on antibacterial activity, with ESC being associated with the least impact [64]. The results of this study provide some support for the antibacterial effect of SSRIs, as there was an overall increase in antibiotic resistance genes. However, it is important to note that this increase was not statistically significant after applying FDR correction. Recent studies have shown that antidepressants can induce antibiotic resistance [38, 65], and in line with this, the expression levels of efflux pump-related genes were elevated in R group. However, this effect was not observed in all individuals, suggesting that the impact of antidepressants on the microbiota may vary between individuals. There were notable differences in the changes in diversity between the R and NR groups, with the former showing a trend towards decreased species richness, but with little significance, and gene richness had recovered to baseline levels within 12 weeks. Functional changes also differed between the two groups, with NR showing a greater functionality decline. This is similar to other studies on antibiotics, which suggest that the recovery of the gut microbiome after antibiotic usage is mediated by specific bacterial species [66]. Typically, alterations in the functionality of the gut microbiota would be anticipated to have an impact on the composition of fecal metabolites. However, in our study, we did not observe significant changes in the fecal metabolome that corresponded to these alterations. Instead, the most notable shifts in fecal metabolites seemed to occur in the NR group over a short period. We speculate that there may be unidentified confounding factors that are impeding these biological changes in fecal metabolites. To confirm this speculation, additional research with more stringent experimental controls is necessary. Collectively, this study suggests that the response of different bacterial groups to the inhibitory effects of ESC may play a role in its efficacy as an antidepressant, and the potential variability in the impact of antidepressants on the gut microbiota between individuals. Further research is needed to delve deeper into these mechanisms and uncover the specific ways by which antidepressants affect the gut microbiota composition, diversity, and function. This knowledge can aid in the development of more targeted approaches, such as personalized probiotic interventions or co-administration of medications that modulate the gut microbiota, to enhance the therapeutic efficacy of antidepressants.

Our integrative analysis yielded insights into the potential significant role played by gut microbiota in the effectiveness of antidepressants. The results obtained from both the procrustes analysis and lasso indicated a stronger connection between blood metabolites and gut microbiota in individuals with MDD, implying that gut microbiota might contribute to depression by influencing blood metabolites. Although our analysis using GLMMLasso revealed associations between specific blood metabolites and gut bacterial species, we cannot yet draw a definitive conclusion regarding the precise biological mechanism underlying the interactions among antidepressants, gut microbiota, and blood metabolites. Nevertheless, the observed associations, especially those involving L-tryptophan and I3PA, present a hypothetical pathway through which antidepressants could potentially impact their efficacy via the gut microbiota. According to this pathway, antidepressants may exert an influence on the gut microbiota, leading to alterations in the tryptophan metabolic pathway, which in turn ameliorates depression. Previous studies have reported that the disturbance of tryptophan metabolism is an essential factor contributing to the pathology of depression [67, 68]. Our results indicated that there was a positively correlation between species richness and I3PA. This further supports the potential involvement of gut microbiota in the pathophysiology of depression through the modulation of tryptophan metabolism.

To analyze the disparities in gut microbiota between remitters (R) and non-remitters (NR) at the start of the study, we employed a multivariate linear regression model. This model took into account various factors known to influence gut microbiota, such as age, gender, BMI, and duration of depression. Although there were statistically significant differences in educational attainment between the R and NR groups, we disregarded it as it was deemed biologically irrelevant to gut microbiota. Additionally, there was a variation in the baseline level of HAMD-17 between the R and NR groups. However, we excluded it from the model due to its high collinearity with the grouping factor. Existing literature indicates a connection between baseline HAMD-17 and remission outcomes for patients [69, 70]. Additionally, considering the minor one-point difference in HAMD-17 scores between the R and NR groups, with both groups falling within the range of moderate severity (a HAMD-17 score above 17 and up to 24 signifies moderate severity), we believe that any systemic bias introduced by excluding it would be negligible. It was found that the R group exhibited higher diversity and a more robust microbial network compared to the NR group. This increased diversity and stronger network structure in the R group suggested that they had better resilience or resistance to the effects of the antidepressant. It is important to note that similar observations of higher diversity providing resistance to drugs or diseases have been reported in other studies [45, 69]. Based on our findings, it is suggested that one potential reason for the higher resistance observed in the R group is the presence of spore formation. Spore formation is a critical mechanism used by bacteria to withstand external disturbances and adverse conditions. Previous studies on fluoxetine have shown that gene expression related to spore formation became more active after intervention [31]. Spore-forming bacteria, such as *E. rectale*, are considered core bacteria in the gut microbiota [70, 71] and our analysis revealed that these bacteria were more abundant in the R group across all three time points. In addition, we also found that spore formation genes were significantly correlated with species richness. Spore-forming bacteria can also promote the 5-HT biosynthesis in colonic enterochromaffin cells (ECs), which provide 5-HT to the mucosa, lumen, and circulating platelets [72]. Based on machine learning models related to spore formation-related genes, their model performance was also better than others and can be used as a potential biomarker for drug efficacy.

Nevertheless, this study has some limitations. First, due to logistical constraints, we were not able to design a longitudinal control group, which may have confounded the effects of time and medication. However, previous studies have shown that the gut microbiota is relatively stable over a short period of time [73, 74]. Second, this was an observational study and we did not strictly control for factors such as patients' lifestyle and diets but rather recorded, which may have introduced confounding variables that influence the observed biological findings to some extent. The grouping of R and NR was based on international standards, which can be subjective to some extent. It is possible that employing more extreme or stringent criteria for grouping could unveil additional differences in treatment efficacy between these groups. Finally, although we mitigate model bias and provide a more robust evaluation of our classification model, it is essential to validate the model in other populations to assess its generalizability.

## Conclusions

In summary, through a longitudinal study involving MDD patients, we conducted a comprehensive analysis of multi-omics data to investigate the role of gut microbiota in remission following ESC treatment. Our analysis of the blood metabolome revealed that ESC treatment ameliorated abnormal blood metabolism by up-regulating MDD-depleted amino acids and down-regulating MDD-enriched fatty acids. Moreover, ESC exerted weak inhibitory effects on the gut microbiota, however, remitters exhibited more resilient microbial community than non-remitters due to higher species richness and sporulation mechanism. Additionally, our findings indicate that the gut microbiota play a role in shaping the variability of the blood metabolome and are associated with several metabolites (e.g., L-tryptophan and I3PA) that are upregulated following ESC treatment. Our data support the notion that an improved gut microbiota profile can contribute to the effectiveness of antidepressant treatment, specifically in the case of ESC. Overall, this study provides new insights into the interconnected relationship between antidepressant medication, blood metabolome alterations, and the gut microbiome in patients with MDD. By shedding light on these mechanisms, our findings have the potential to enhance our understanding of how ESC exerts its therapeutic effects in alleviating depression. Furthermore, this knowledge can aid in the development of improved treatment strategies for MDD by targeting the gut microbiota and modulating the associated blood metabolome.

## Materials and methods

### Study design

A total of 276 individuals (aged 18 to 65) were selected, including 166 HCs and 110 MDD patients. The patients included in the study were required to meet the Diagnostic and Statistical Manual of Mental Disorders, 4th Edition (DSM-IV) criteria for major depressive disorder (MDD). The diagnosis was determined based on the Chinese version of the Mini-International Neuropsychiatric Interview (MINI), which was consistent with the methodology used in our previous studies [21, 22]. HCs were recruited through advertisements, and were also screened using the MINI to ensure that they did not meet any of the criteria for DSM-IV Axis I psychiatric disorders. The exclusion criteria were as follows: (i) individuals with pre-existing conditions such as diabetes, thyroid disease, cardiovascular disease, or cancer; (ii) alcohol or drug abuse, and/or acute intoxication; (iii) a history of bipolar disorder, schizophrenia, schizoaffective disorder, or other Axis I psychiatric disorders; (iv) pregnancy or breastfeeding; (v) a history of antibiotic use or changes in diet habits within one month prior to sampling. The study protocol was approved by the Human Research and Ethics Committee of Beijing Anding Hospital (No. 2017-24), Capital Medical University (China). All participants provided written informed consent prior to the commencement of the study.

#### Treatment and measurements

All MDD patients were treated with ESC for a period of 12 weeks. Previous studies have confirmed the efficacy and acceptability of ESC, which is one of the most commonly prescribed medications for patients with MDD in Asian countries [75–77]. According to the clinical practice guidelines, the dosing regimen for ESC in this study involved a titration process. The initial dose of ESC was set at 5 mg per day, and within a period of 7 days, it was gradually increased to a range of 10–20 mg per day. Once the optimal dose was determined, it remained stable for the duration of the 12-week trial [78]. No other medications were allowed during the study period. The HAMD-17 was used to assess the severity of MDD, which was rated by experienced and trained independent raters. All patients were evaluated at baseline and after 12 weeks of ESC treatment. The Diet and Lifestyle Questionnaire was used to assess participants' habitual dietary intake and lifestyle (Table S11).

#### Metagenomic sequencing of fecal samples

DNA extraction, library construction and metagenomic sequencing of the fecal samples were performed as described previously [11]. Total genomic DNA was extracted from stool samples using the E.Z.N.A. Soil DNA Kit (Omega Bio−tek, Norcross, GA, USA) according to the manufacturer’s protocol. After genomic DNA extraction, the concentration and purity of DNA samples were determined using TBS−380 and NanoDrop2000, respectively. DNA integrity was detected using 1% agarose gel electrophoresis. The DNA extract was fragmented by Covaris M220 (Gene Company Limited, China) and the resulting fragments (approximately 300 bp) were screened and used to construct paired−end libraries. The paired−end library was then constructed using NEXTFLEX ^®^ Rapid DNA−Seq (Bioo Scientific, Austin, TX, USA). Adapters containing the full complement of sequencing primer hybridization sites were ligated to the blunt−end of fragments. Paired−end sequencing was performed using the Illumina NovaSeq platform (Illumina Inc., San Diego, CA, USA) according to manufacturer’s instructions (www.illumina.com) at Shanghai Majorbio Bio−pharm Technology Co. Ltd.

#### Gas chromatography—mass spectrometry

The fecal samples and plasma samples were prepared for metabolite extraction and derivatization pre-treatment. The derivatives were stored at ambient temperature for 30 min, and then analyzed using the Agilent 7890A-5975C GC–MS platform (Agilent, USA). The derivatized samples were injected into the GC–MS system in non-split mode with an injection volume of 1 μL. The samples were separated on an HP-5MS capillary column (30 m × 0.25 mm × 0.25 μm, Agilent J&W Scientific, Folsom, CA, USA), and then detected by mass spectrometry. The data were acquired in a full-scan mode (m/z 50–600). To avoid the effects caused by fluctuations in the instrument signal, a random order was used for continuous sample analysis. The raw data obtained from GC–MS were pre-processed by ChromaTOF software (v 4.34, LECO, St Joseph, MI), and the three-dimensional matrices were exported in a CSV format. The response intensity of the mass spectrometry peaks was then normalized. The integrated data matrix was imported into SIMCA-P + 14.0 software package (Umetrics, Umeå, Sweden), and unsupervised principal component analysis (PCA) was performed to determine the overall distribution among the samples and the stability of the whole analysis process. Then, supervised (orthogonal) partial least squares analysis (OPLS-DA) was conducted to identify the discriminant metabolites with the significance threshold of variable importance plot (VIP)å 1.0 and *P* < 0.05. The OPLS-DA models were validated by permutation analysis (200 times).

#### Metagenomic data

Raw paired-end reads were initially trimmed and filtered using Trimmomatic (v0.39) [79]. The trimmed reads were then host-filtered using BWA MEM (v0.7.17-r1188) [80]. Metagenome assembly was processed by MEGAHIT (v1.1.3) with default parameters [81]. The sequence data of each sample were assembled separately. Genes present in the assembled contigs were identified using MetaGeneMark [82], and the redundant genes were then removed (CD-HIT) [83], resulting in a non-redundant microbial gene catalogue of 8,568,218 genes. Taxonomic assessment of genes was performed using a fast LCA algorithm implemented in SqueezeMeta [84]. This database searches for the last common ancestor of the hits for each query gene based on the results of the Diamond search against the GenBank nr database. For functional annotation, the eggNOG was annotated by aligning genes to eggNOG database using eggnog-mapper (v2.1.3) [85]. Antibiotic resistance genes (ARGs) were annotated by alignment against the Comprehensive Antibiotic Resistance Database (CARD) using RGI (v5.1.1) [86]. A total of 66 sporulation signature genes were obtained from previous research [87]. Heuristic based bidirectional best hit analysis was performed to detect potential sporulation genes in non-redundant gene catalogue using BLAST.

To estimate the abundance of sporulation genes in each sample, high quality reads were mapped onto the non-redundant gene catalogue. Custom scripts were used to quantify their relative abundance using reads/kilobase/million mapped reads (RPKM). The abundances of KEGG orthology (KO), KEGG pathway, eggNOG orthology, Antibiotic Resistance Ontology (ARO), and sporulation genes were calculated by summing the abundances of all the items falling within each category.

Taxonomic profiling was performed on the high-quality reads using MetaPhlAn3 against the mpa_v30_CHOCOPhlAn_201901 database [88]. Functional read profiling was performed using HUMAnN3 [88]. Low-abundance filters were applied to eliminate taxonomic and functional features that were present in less than 0.1% and 0.001% of the total abundance, respectively, among at least 10% of the individuals. To address compositional effects in microbiome datasets, the centered log ratio (CLR) transformation was performed on our taxonomic and functional data.

Microbiome data dissimilarity index was used to estimate between-sample diversity (beta diversity) based on the species-level relative abundance profiles. Diversity metrics and ordinations were performed using the phyloseq package [89]. Several diversity indexes were used to estimate species diversity within individual metagenomic samples (alpha diversity). The Bray–Curtis microbiome composition was visualized by Principal Coordination Analysis (PCoA).

#### Metabolomic data

Only metabolites present in > 10% of the samples were analyzed. The metabolites were normalized by log transformation, and then scaled using the Pareto scaling method. Pareto scaling involves dividing each variable by the square root of its standard deviation. It is able to reduce the weight of large fold changes in metabolite signals, which is more significant than auto scaling [90].

#### Statistical analysis

Permutational Multivariate Analysis of Variance (PERMANOVA) tests (4,999 permutations and “adonis2” function in the “vegan” package) were employed to assess the overall effect of ESC treatment on multi-omics data. For the longitudinal data, PERMANOVA was implemented using a mixed-model design. Individual identity was included as a blocking factor (“strata”) to control for repeated sampling. To assess between-sample variation, Euclidean distance was used for the metabolome, while Bray–Curtis distance for the microbiome.

Longitudinal changes in omics features (including microbial diversity/other indices, microbial taxa, microbial functions, plasma metabolites and fecal metabolites) across different time points were assessed using linear mixed model (LMM). The model included age, sex, BMI, MDD duration and visit week as fixed effects, and subject ID as the random effect [omics feature ~ age + sex + BMI + MDD duration + visit week + (1 | subject ID)]. We also repeated this analysis using MaAsLin2, which supports multivariable association analysis with repeated measures in longitudinal data. Two models were further used to assess differences between the remitter (R) and non-remitter (NR) groups after ESC treatment (adjusted by baseline abundance) [omics feature ~ age + gender + BMI + MDD duration + baseline + group + (1 | subject ID)], and the difference in change (slope) from baseline to week 12 between the R and NR groups, with the interaction term visit week-by-group [omics feature ~ age + sex + BMI + MDD duration + visit week * group + (1 | subject ID)].

The association between each individual feature and remission group at the three time points was estimated using multivariate linear regression. To correct for confounding, the regression model also included age, sex, BMI and MDD duration. CLR transformation was conducted on the microbiome data to break the compositionality of the data and normalize skewed distributions of microbiome features. The Benjamini–Hochberg correction was used to control for multiple testing. All data were considered significant at FDR < 0.1, unless specifically stated. All statistical analyses and visualization were performed in R (version 4.2.0).

#### Integration analysis with Lasso and glmmLasso

Lasso penalized regression was used to determine the associations between metabolites and microbial taxa in both HC and MDD cohorts, as illustrated in gene-microbiome and serum metabolomic-microbiome [41, 91]. Briefly, the metabolite-based LASSO model employed the scaled abundance of each metabolite as a response and the CLR-transformed abundance of microbiome taxa as predictors to identify the metabolites associated with microbial taxa. The model leveraged leave-one-out cross-validation to estimate the optimization parameter λ, which was used to fit the final model on the cohort dataset. R package glmnet (version 2.0–13) was used to implement the lasso regression model (version 2.0–13) [92]. The lasso model was then inferred using a regularized projection method known as declassified lasso. This approach employs the R package "hdi" to calculate 95% confidence intervals and *P* values for the coefficients associated with each microbe that exhibited an association with a specific metabolite. The Benjamini-Hochberg (FDR) method was used to correct for multiple hypothesis testing.

To establish a connection between longitudinal plasma metabolites and gut microbiota, the mixed effects least absolute shrinkage and selection operator (mixed effects LASSO) approach was conducted using the glmmLasso function in the R package glmmLasso [93]. The specific model used for this analysis is as follows: metabolite ~ taxa1 + taxa2 + … + taxaN + age + gender + BMI + drug use + (1|subject ID). The best lambda was selected from a lambda loop from 0 to 200 and incremented by 1 as the smallest model BIC. To ensure robustness and account for potential sampling variability, the model was repeated 100 times. In each iteration, a random sample comprising 70% of the total samples was used. Finally, the linear mixed model was re-fitted specifically for the features that had non-zero mean coefficients in order to determine their significance.

#### Random forest with nested cross-validation

To assess the predictive potential of multi-omics features for remission prediction, a random forest algorithm was performed on the microbial taxa and function profiles using the R package caret [94]. To overcome performance bias and ensure reliable model selection, a nested cross-validation approach was employed. This technique combines both model selection and hyperparameter optimization procedures. The outer loop serves for assessing the quality of the model, while the inner loop serves for model/parameter selection. In our case, the outer loop was repeated 10 times, resulting in 10 different test sets. For each iteration, feature selection, as performed by mRMR [95], was implemented in the outer train set, and the best model was selected in the inner layer via leave-one-out cross-validation (LOOCV). The area under the curve (AUC) was used to measure the model’s performance.

### Supplementary Information

**Additional file 1: Fig. S1.**Changes in plasma metabolome/metabolites after ESC intervention. a Principal coordinate analysis (PCA) plots based on the plasma metabolic signatures of MDD subjects at baseline, week 2 and week 12. b Post-treatment difference in serotonin between the R and NR groups after adjustment for baseline. c Longitudinal changes in cholic acid and chenodeoxycholic acid, which exhibited differential trends between the R and NR groups. The P values were obtained from LMMs applied to grouped data of R and NR. **Fig. S2.** Effects of ESC intervention on the gut microbiome. a Changes in microbial Shannon at species and gene level from baseline to week 12 in ALL, R and NR groups. ALL included the samples from both R and NR groups, Shannon of HC was used as the reference value. The table presents detailed results from longitudinal comparisons using LMMs, with coefficients and P values. b Scatter plot showing a significant negative correlation (spearman) between the species richness and DS at three visit weeks. c Heatmap of altered sporulation genes at week 2 and week 12 after ESC intervention in ALL, R and NR groups, as determined by LMMs. The coefficients have different colors of grey (high) and red (low). Significant differences are indicated by * *p* < 0.05, *** q < 0.1. d Longitudinal changes in Firmicutes from baseline to week 12 in ALL, R and NR groups after ESC intervention, and its abundance was significantly increased in the ALL and NR groups from baseline to week 2. e Scatter plot showing the spearman's correlations between visit weeks (delta) of Firmicutes and species richness. f Plot revealing the effects of visit week × group interaction on the four species. The P values were derived from LMMs involving visit week x group interaction. **Fig. S3.** Integration analysis results of fecal metabolome and gut microbiome. a Multiple dimensional scaling (MDS) plot of procrustes analysis showing overall association between fecal metabolome and gut microbiome in MDD and HC cohorts, with individual samples being connected by a line. Euclidean distance was used for fecal metabolome (blue circles), while Bray-Curtis distance for gut microbiome data (red circles), and the procrustes m2 statistic results were labeled. b Venn diagram showing few overlaps of association between plasma metabolites and microbial species. Only 6 microbiota-associated plasma metabolites were shared between the MDD and HC cohorts. **Fig. S4.** Microbiome comparison between the R and NR groups. a The spearman’s correlation between the species richness and HAMD-17 scores at baseline. b Differences in the Shannon values of microbial species (left) and gene (right) between the R and NR groups at baseline, with HC as reference. c Species co-abundance network of the R and NR groups constructed by SparCC. The community structure and network in R group was more complex and well-organized than those in NR group at baseline (only coefficients > 0.5 and coefficient < -0.5 are displayed), Color represents phylum level. d Natural connectivity to assess the robustness of microbial ecological interaction networks for sequential node removal. The order of node removal was random or ordered by degree or betweenness centrality. Natural connectivity is shown as a function of the relative size of the network. e Boxplot of the differential abundance of Eubacterium rectale between the R and NR groups at three visit weeks. f The spearman’s correlation between the species richness and abundance of E. rectale at three visit weeks. **Fig. S5.** Nest cross-validation schematic diagram for the construction of prediction models. a For each dataset, the samples were divided into train set, validation set and test set. Nested cross validation was composed by two loops: outer loop and inner loop. The outer loop serves for assessing the quality of the model, while the inner loop serves for model/parameter selection. The outer loop was repeated 10 times, resulting in 10 different test sets. For each iteration, feature selection was implemented in the outer train set, and the best model was selected in the inner layer via leave-one-out-cross-validation (LOOCV). The area under curve (AUC) was used to measure model’s performance. b The combined ROC curve of 10 outer loop testing results obtained from RF model based on the changes in sporulation genes from baseline to week 2.)

**Additional file 2: Table S1.** Demographics and clinical characteristics between MDD and HC. **Table S2. **Demographics and clinical characteristics between R and NR.

**Additional file 3: Table S3.** The influence of confounding factors on bacterial composition between MDD and HC. **Table S4.** The influence of confounding factors on bacterial composition between R and NR.

**Additional file 4: Table S5.** Changes in antimicrobial resistance (AMR) genes in the ALL, R and NR groups after ESC treatment

**Additional file 5: Table S6.** Changes in fecal metabolites in the ALL, R and NR groups after ESC treatment

**Additional file 6: Table S7.** Significant plasma metabolite-microbe associations at FDR < 0.1 for HC data (results from Lasso). **Table S8.** Significant plasma metabolite-microbe associations at FDR < 0.1 for MDD data at baseline (results from Lasso).

**Additional file 7: Table S9.** Significant plasma metabolite-microbe associations at FDR < 0.1 for all MDD sample data(results from LMM, with species variables selected by GLMMLasso)

**Additional file 8: Table S10.** Spearman's correlations between richness index and sporulation genes at FDR < 0.05 at baseline

**Additional file 9: Table S11**. Diet and Lifestyle Questionnaire

## Data Availability

All data required to evaluate the conclusions of the paper are available in the paper and/or the Supplementary Materials. The metagenomic sequencing data has been deposited in the China National GeneBank DataBase (CNGBdb) (https://db.cngb.org/; project ID: CNP0004519). The data of fecal and blood metabolomics has been deposited in the MetaboLights repository (MTBLS8112). Additional data related to this paper can be requested from the authors.
