# Supplementary material for: Multi-omics reveal microbial determinants impacting the treatment outcome of antidepressants in major depressive disorder
Source: Microbiome. 2023 Aug 28;11:195. doi: 10.1186/s40168-023-01635-6 (PMC10464022; doi:10.1186/s40168-023-01635-6)
Supplement: Supplementary file 2 — Additional file 1: Fig. S1.Changes in plasma metabolome/metabolites after ESC intervention. a Principal coordinate analysis (PCA) plots based on the plasma metabolic signatures of MDD subjects at baseline, week 2 and week 12. b Post-treatment difference in serotonin between the R and NR groups after adjustment for baseline. c Longitudinal changes in cholic acid and chenodeoxycholic acid, which exhibited differential trends between the R and NR groups. The P values were obtained from LMMs applied to grouped data of R and NR. Fig. S2. Effects of ESC intervention on the gut microbiome. a Changes in microbial Shannon at species and gene level from baseline to week 12 in ALL, R and NR groups. ALL included the samples from both R and NR groups, Shannon of HC was used as the reference value. The table presents detailed results from longitudinal comparisons using LMMs, with coefficients and P values. b Scatter plot showing a significant negative correlation (spearman) between the species richness and DS at three visit weeks. c Heatmap of altered sporulation genes at week 2 and week 12 after ESC intervention in ALL, R and NR groups, as determined by LMMs. The coefficients have different colors of grey (high) and red (low). Significant differences are indicated by * p < 0.05, *** q < 0.1. d Longitudinal changes in Firmicutes from baseline to week 12 in ALL, R and NR groups after ESC intervention, and its abundance was significantly increased in the ALL and NR groups from baseline to week 2. e Scatter plot showing the spearman's correlations between visit weeks (delta) of Firmicutes and species richness. f Plot revealing the effects of visit week × group interaction on the four species. The P values were derived from LMMs involving visit week x group interaction. Fig. S3. Integration analysis results of fecal metabolome and gut microbiome. a Multiple dimensional scaling (MDS) plot of procrustes analysis showing overall association between fecal metabolome and gut microbiome in [file 40168_2023_1635_MOESM1_ESM.docx]

**Figure Legends**


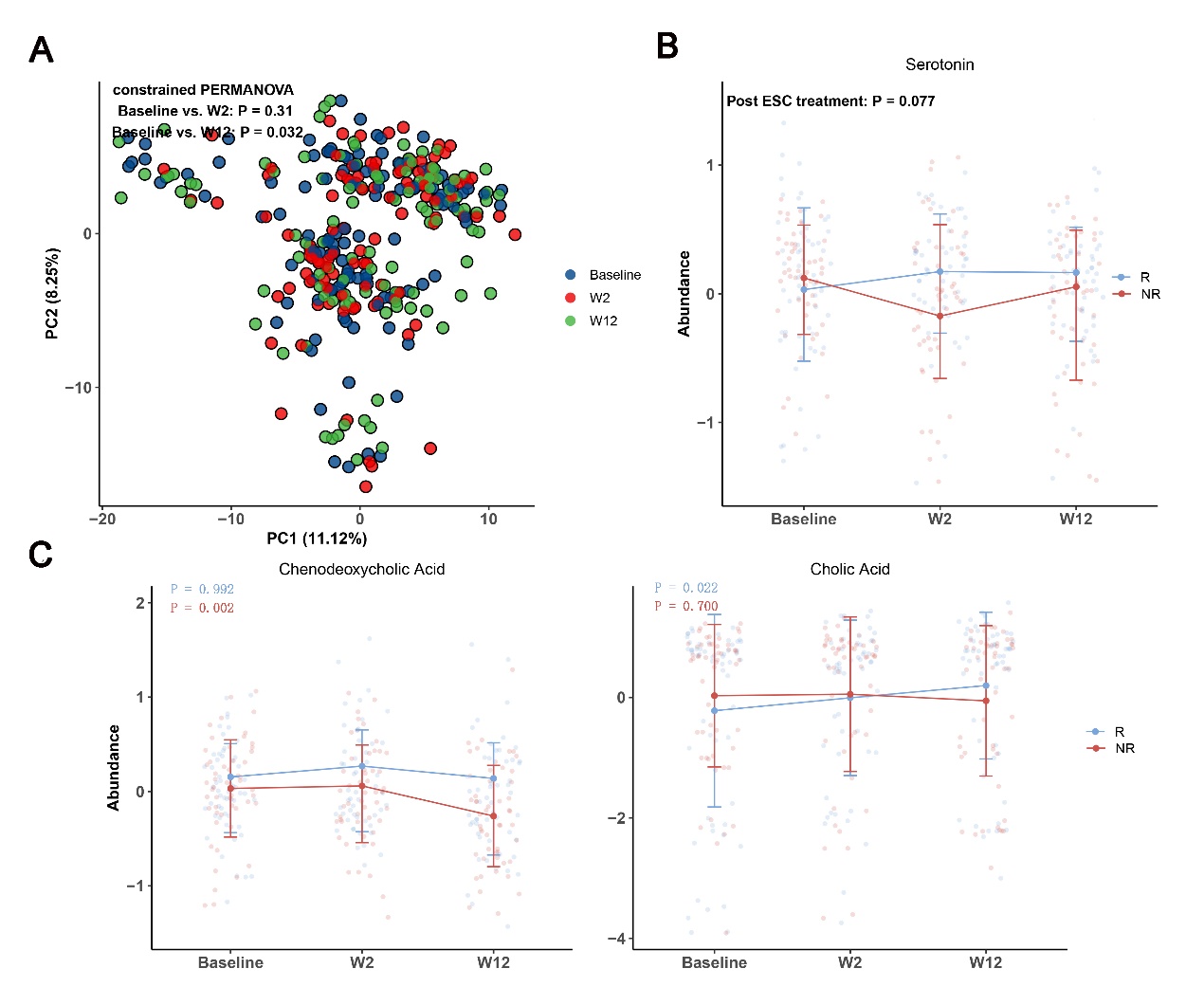


**Fig. S1** Changes in plasma metabolome/metabolites after ESC intervention. **a** Principal coordinate analysis (PCA) plots based on the plasma metabolic signatures of MDD subjects at baseline, week 2 and week 12. **b** Post-treatment difference in serotonin between the R and NR groups after adjustment for baseline. **c** Longitudinal changes in cholic acid and chenodeoxycholic acid, which exhibited differential trends between the R and NR groups. The P values were obtained from LMMs applied to grouped data of R and NR.


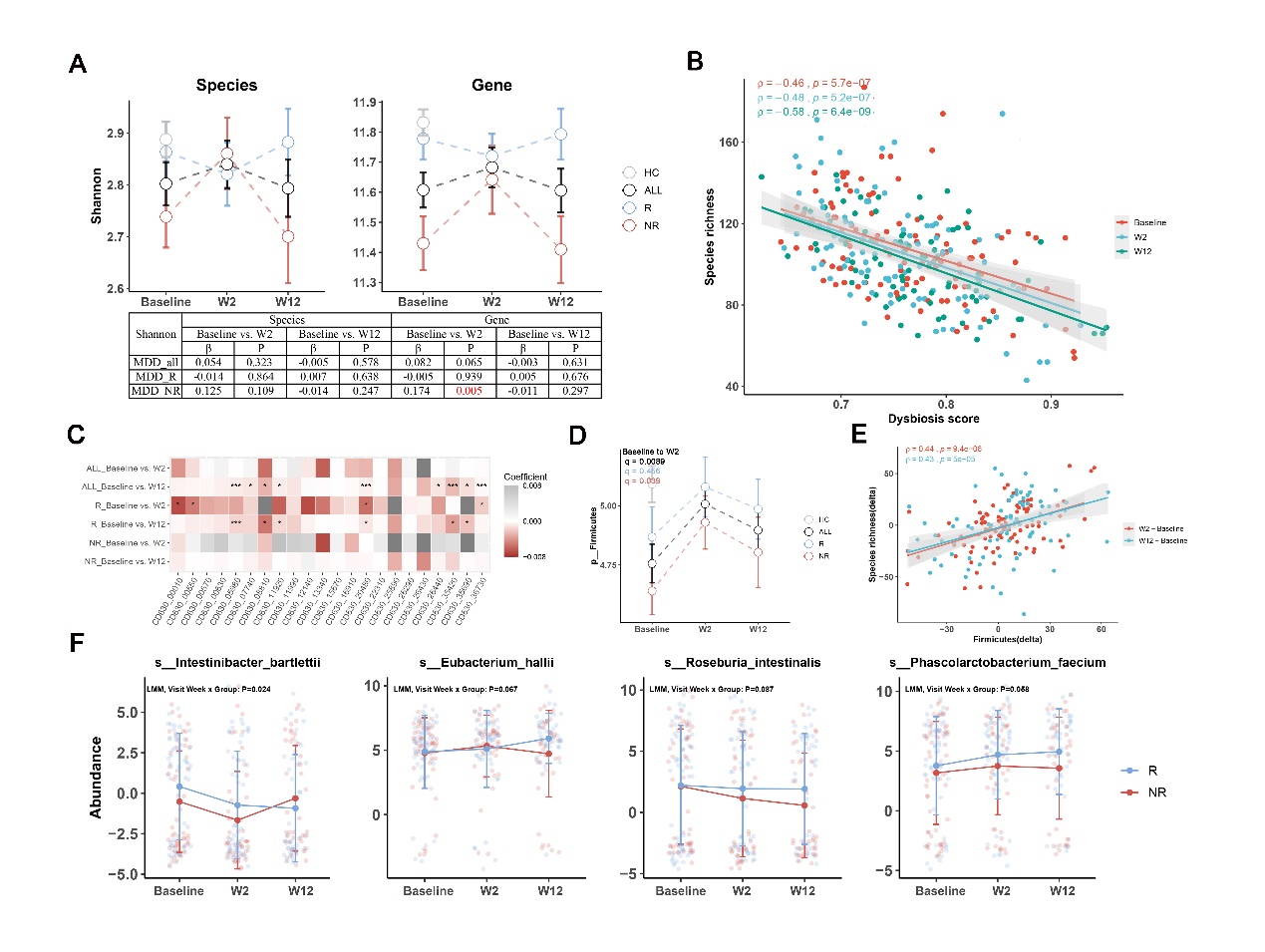


**Fig. S2** Effects of ESC intervention on the gut microbiome. **a** Changes in microbial Shannon at species and gene level from baseline to week 12 in ALL, R and NR groups. ALL included the samples from both R and NR groups, Shannon of HC was used as the reference value. The table presents detailed results from longitudinal comparisons using LMMs, with coefficients and P values. **b** Scatter plot showing a significant negative correlation (spearman) between the species richness and DS at three visit weeks. **c** Heatmap of altered sporulation genes at week 2 and week 12 after ESC intervention in ALL, R and NR groups, as determined by LMMs. The coefficients have different colors of grey (high) and red (low). Significant differences are indicated by * p < 0.05, *** q < 0.1. **d** Longitudinal changes in Firmicutes from baseline to week 12 in ALL, R and NR groups after ESC intervention, and its abundance was significantly increased in the ALL and NR groups from baseline to week 2. **e** Scatter plot showing the spearman's correlations between visit weeks (delta) of Firmicutes and species richness. **f** Plot revealing the effects of visit week × group interaction on the four species. The P values were derived from LMMs involving visit week x group interaction.


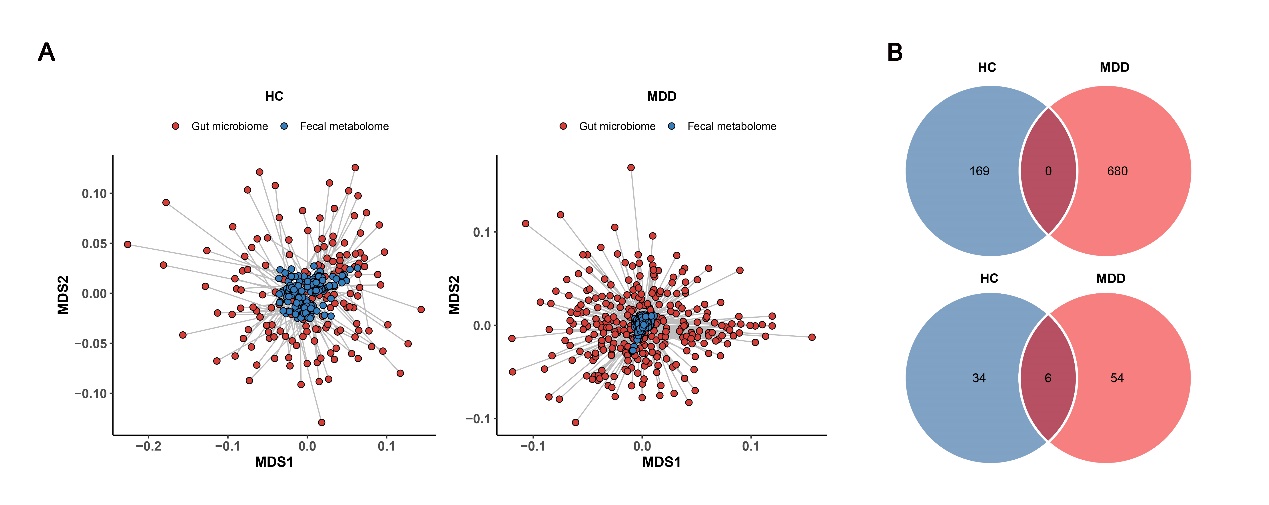


**Fig. S3** Integration analysis results of fecal metabolome and gut microbiome. **a** Multiple dimensional scaling (MDS) plot of procrustes analysis showing overall association between fecal metabolome and gut microbiome in MDD and HC cohorts, with individual samples being connected by a line. Euclidean distance was used for fecal metabolome (blue circles), while Bray-Curtis distance for gut microbiome data (red circles), and the procrustes m^2^ statistic results were labeled. **b** Venn diagram showing few overlaps of association between plasma metabolites and microbial species. Only 6 microbiota-associated plasma metabolites were shared between the MDD and HC cohorts.


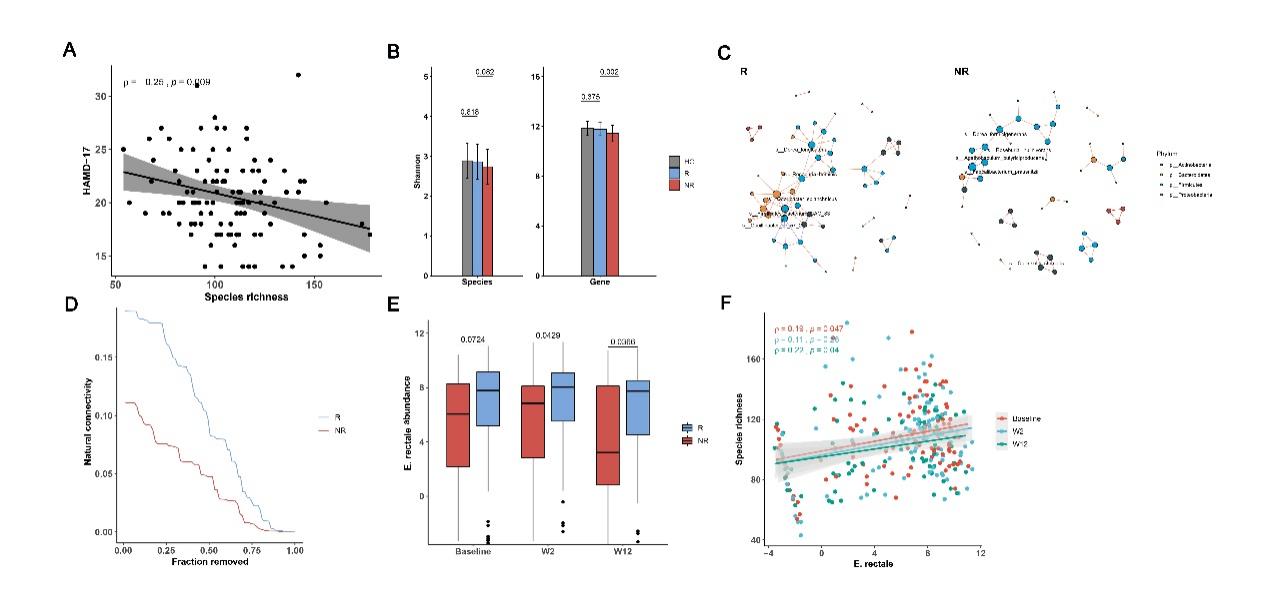


**Fig. S4** Microbiome comparison between the R and NR groups. **a** The spearman’s correlation between the species richness and HAMD-17 scores at baseline. **b** Differences in the Shannon values of microbial species (left) and gene (right) between the R and NR groups at baseline, with HC as reference. **c** Species co-abundance network of the R and NR groups constructed by SparCC. The community structure and network in R group was more complex and well-organized than those in NR group at baseline (only coefficients > 0.5 and coefficient < -0.5 are displayed), Color represents phylum level. **d** Natural connectivity to assess the robustness of microbial ecological interaction networks for sequential node removal. The order of node removal was random or ordered by degree or betweenness centrality. Natural connectivity is shown as a function of the relative size of the network. **e** Boxplot of the differential abundance of *Eubacterium rectale* between the R and NR groups at three visit weeks. **f** The spearman’s correlation between the species richness and abundance of *E. rectale* at three visit weeks.


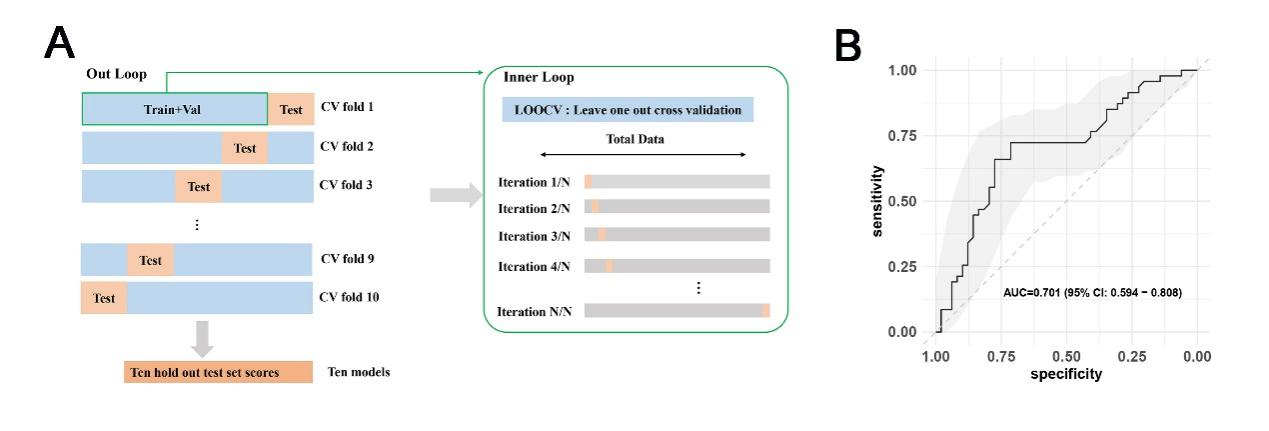


**Fig. S5** Nest cross-validation schematic diagram for the construction of prediction models. **a** For each dataset, the samples were divided into train set, validation set and test set. Nested cross validation was composed by two loops: outer loop and inner loop. The outer loop serves for assessing the quality of the model, while the inner loop serves for model/parameter selection. The outer loop was repeated 10 times, resulting in 10 different test sets. For each iteration, feature selection was implemented in the outer train set, and the best model was selected in the inner layer via leave-one-out-cross-validation (LOOCV). The area under curve (AUC) was used to measure model’s performance. **b** The combined ROC curve of 10 outer loop testing results obtained from RF model based on the changes in sporulation genes from baseline to week 2.
